# Restoring CFAR Validity for Single-Channel IoT Sensor Streams: A Monte Carlo Comparison of CFAR-Family and Sequential Detectors Under Cortex-M0+ Constraints

**DOI:** 10.3390/s26175543

**Published:** 2026-08-31

**Authors:** Sergii Makovetskyi, Oleksii Zhelanov, Viktor Kauk, Lars Thomsen

**Affiliations:** 1 Department of Software Engineering, Kharkiv National University of Radio Electronics, 61166 Kharkiv, Ukraine; 2 Department of Media Engineering and Information Radio Electronic Systems, Kharkiv National University of Radio Electronics, 61166 Kharkiv, Ukraine; 3 Gnacode Inc., Medicine Hat, AB T1A 0J5, Canada

**Keywords:** adaptive thresholding, CFAR, CA-CFAR, OS-CFAR, CUSUM, edge computing, event detection, IoT sensor networks, mesh networks, Monte Carlo simulation

## Abstract

Real-time event detection in IoT mesh sensor networks must balance sensitivity against the false-positive load placed on a constrained mesh radio. We present a Monte Carlo comparison of the Temporal Spectral Noise-Floor Adaptation (TSNFA) detector against classical comparators drawn from the radar Constant False Alarm Rate (CFAR) family and from sequential change detection: the Lipski FFT energy detector, Cell-Averaging CFAR (CA-CFAR), Ordered-Statistic CFAR (OS-CFAR), and state-machine Cumulative Sum (CUSUM), with four further family members (GO, SO, TM, VI) measured in one additional configuration. All detectors fit a Cortex-M0+ class envelope, process a 1-D 100 Hz time series in 128-sample frames, and use temporal reference windows in place of spatial reference cells. The simulation covers nine SNR levels (1.5 to 24 dB) at 10 nodes with scale checks at 50 nodes, each test configuration replicated five times over 24 h. The recommended TSNFA configuration, selected by an estimator ablation, combines a mean trigger statistic, a gated median noise floor, and a three-frame confirmation rule. TSNFA detects 100% of events at 12 dB SNR and above, 99.5% at 5 dB, and 80.3% at 3 dB, with 99.6–100% event precision and 0.0017 false-positive clusters per node hour at a cost of 2.6 s of added detection latency from confirmation. CA-CFAR and OS-CFAR detect every event but produce 42.6 and 36.8 false-positive clusters per node hour; the Lipski detector reaches 1.24 clusters per node hour at 41–45% precision but detects no events below 6 dB; CUSUM detects 16–72% of events across the SNR range. TSNFA is the only detector tested that combines high detection rate, high precision, and low false-positive load within the Cortex-M0+ envelope.

## 1. Introduction

IoT sensor networks for perimeter security, structural health monitoring, environmental sensing, and industrial process control share a common architectural problem. Each node observes a continuous time series, must autonomously decide when something of interest has occurred, and must transmit only those decisions across a constrained mesh radio link. The decision rule sits at the intersection of two budgets that pull in opposite directions: detection must be sensitive enough that genuine events are not missed, yet selective enough that the network is not flooded by false positives. A missed event compromises the monitoring function; a false positive consumes bandwidth, drains battery, and, in dense mesh networks, can cascade through relay paths and saturate the sink. Repeated false positives also erode operator trust in the detection output, to the point where alarms are ignored or nodes are muted. Reducing the data volume that leaves the node is a recognized design goal for edge IoT systems in its own right [1,2].

The radar community addressed a closely related problem more than 50 years ago with the family of Constant False Alarm Rate (CFAR) detectors. Finn and Johnson [3] introduced Cell-Averaging CFAR (CA-CFAR) in 1968, in which the threshold for a cell under test is computed from a sliding mean of nearby reference cells. Rohling [4] introduced Ordered-Statistic CFAR (OS-CFAR) in 1983, replacing the mean with a rank-order statistic for robustness against interfering targets. Subsequent work produced a broad family of variants, including greatest-of and smallest-of selection logic [5,6], censored and trimmed-mean estimators [7,8,9], and variability index-switching schemes [10,11]. The defining property of all CFAR detectors is that the threshold is recomputed from local data, so that the false alarm probability remains approximately constant as the underlying noise statistics drift. Section 2 reviews this family in detail and states the criteria used to select the comparators evaluated here.

TSNFA, proposed by Makovetskyi and Thomsen [12], can be read as a CFAR detector adapted to two constraints the radar formulation does not face. First, the input is a 1D temporal stream rather than a 2D range-Doppler map; the reference window must be drawn from previous frames of the same node. Second, the hardware envelope is far more constrained than a radar receiver. The deployed implementation runs on an STM32G030F6 (Arm Cortex-M0+ at 8 MHz, no hardware FPU, 32 kB Flash, 8 kB SRAM) and must complete a detection cycle within the 1.28 s frame period while leaving spare cycles for radio communication; the measured resource costs of all implemented detectors are reported in Section 3.5. TSNFA keeps the CFAR core, a threshold computed from a local statistic on a reference window, and adds three defenses: band selection (out-of-band interference removed before thresholding), temporal persistence (single-frame in-band transients outvoted), and three-frame confirmation (binary integration in the sense of Schwartz) [13]. A second relevant class is sequential change detection. The Cumulative Sum (CUSUM) statistic [14] detects an abrupt change in distribution by accumulating log-likelihood ratio increments. We adopted the state-machine variant of Torre et al. [15], whose clipped score prevents accumulator overflow in fixed-precision arithmetic, with the Tartakovsky linear-quadratic instantaneous log-likelihood ratio [16] as the per-sample increment. A third class is machine-learning anomaly detection on microcontrollers, now an active field with several surveys [17,18,19,20,21] and deployed systems [22,23,24], alongside neural-network and knowledge-based threshold stages within the CFAR family itself [25,26,27,28]. These methods are excluded from the present comparison on a compute criterion: their inference cost is one to two orders of magnitude above the classical CFAR family, and the lightest reported deployments occupy on the order of 80 kB of RAM on ESP32-class hardware [23] against 8 kB of total SRAM on the target platform (per-frame cycle counts for the implemented detectors are measured in Section 3.5). A fair comparison belongs to Cortex-M4 or M7 class hardware. Section 2 details this exclusion tier.

The contribution of the present paper is focused on the resource-constrained class of detectors that can run on a Cortex-M0+. We compare TSNFA against the four most natural classical comparators (Lipski FFT [29], CA-CFAR, OS-CFAR, CUSUM), selected by the structural criteria of Section 2, on a Monte Carlo simulation of a perimeter-security signal scenario. All five detectors are implemented under identical hardware constraints (1D temporal stream, 128-sample frames, temporal reference window, no spatial cells) and run at their canonical published parameters. The simulation covers nine SNR levels from 1.5 to 24 dB at a network size of 10 nodes, with scale checks at 50 nodes, and one additional configuration measuring four further CFAR family members (GO, SO, TM, VI) at canonical parameters. Each test configuration is replicated five times for variance estimation over 24 h of simulated operation.

In summary, the main findings are that the recommended hybrid TSNFA configuration (mean trigger statistic, gated median noise floor, three-frame confirmation) detects 100% of events at 12 dB SNR and above and 80.3% at 3 dB, with 99.6–100% event precision and 0.0017 false-positive clusters per node hour, and that no classical CFAR family or sequential change-detection comparator matches this combination: broadband CA-CFAR and OS-CFAR fail on precision, the Lipski FFT detector makes virtually no detections below 6 dB SNR, and CUSUM misses up to 84% of events at low SNR. Two implications follow. Computing a per-bin spectral magnitude at the FFT step restores the chi-squared (exponential) cell statistic that textbook CFAR calibration assumes and that broadband with no spectral selectivity single-node adaptations violate. Detector compute energy on the target microcontroller is orders of magnitude below radio transmit energy, so false-positive suppression, not code optimisation, determines node battery life in mesh deployments.

## 2. Background and Theoretical Setting

### 2.1. The CFAR Family and Reference Cells

The CFAR detector, in its original radar formulation [3,4], operates on a 2D range-Doppler matrix. For each cell under test, the detection threshold is *T* = *a* · *Z*, where:

- *Z* is an estimator of the local clutter level, computed from *N* neighbouring reference cells;
- *N* is the number of reference cells, with guard cells separating the reference window from the cell under test;
- *a* is the threshold multiplier, chosen to achieve a target false alarm probability;
- *P_fa_* is the design false alarm probability per cell test.

CA-CFAR uses the arithmetic means as Z; OS-CFAR uses the k-th order statistic. In all variants, the reference window is spatial: measurements made at the same time instant but at different range or Doppler positions.

Later variants refine the estimator Z against nonhomogeneous reference windows. In the mean-level branch, GO-CFAR takes the greater of the leading and lagging half-window means to control false alarms at clutter edges [6], and SO-CFAR takes the smaller to protect detection of closely spaced targets [5]. In the ordered-statistics branch, TM-CFAR trims both ends of the sorted reference window before averaging [7], and the censored-mean detectors CMLD [8] and GCMLD [9] discard the largest reference cells to resist interfering targets; CMLD is the special case of TM-CFAR that trims from the top only. The switching branch selects among estimators at run time: VI-CFAR computes a variability index and a mean ratio on the half windows and switches among CA, GO, and SO logic accordingly [10], and RVI-CFAR extends the scheme with multi-stage refinements against interferer placement [11]. A more recent line of work inserts learned components into the threshold stage: clutter-knowledge CFAR conditions the threshold on a classifier output [25], and neural-network detectors replace or post-process the threshold comparison [26,27,28]. CFAR detectors have also been transposed out of radar, to sonar [30], and to millimeter-wave traffic radar with Monte Carlo threshold estimation [31], but the cost of transposition for the cell-distribution assumption examined in Section 2.3 has received little attention.

### 2.2. Comparator Selection and Exclusion Criteria

The comparison set is selected by three criteria: (i) structural comparability, meaning the detector operates on a 1D temporal reference window with a single event class, so the comparison isolates the threshold architecture rather than the reference geometry; (ii) canonical parameters, meaning published parameter values exist, so the comparison measures the published detector; and (iii) compute envelope, meaning the detector fits the Cortex-M0+ resource budget of Section 3.5. These criteria produce three tiers. Tier 1 detectors are evaluated across the full configuration matrix: TSNFA, the Lipski FFT energy detector, CA-CFAR, OS-CFAR, and CUSUM. Tier 2 detectors are evaluated at canonical parameters in one additional configuration (10 nodes, 12 dB): GO-CFAR, SO-CFAR, TM-CFAR, and VI-CFAR, as the representatives of the greatest-of, smallest-of, trimmed, and switching branches. Tier 3 detectors are cited, but not simulated, on two grounds. The censoring and switching refinements, CMLD, GCMLD, and RVI, interpolate between behaviours bracketed by tier 2: CMLD is a one-sided special case of TM-CFAR, and the failure modes these refinements address, clutter edges and multiple interfering targets in a 2D reference window, do not arise in a 1D temporal window with a single event class. The learned-component detectors [25,26,27,28] and microcontroller machine-learning pipelines [17,18,19,20,21,22,23,24] exceed criterion (iii): their inference cost is one to two orders of magnitude above the classical family, and the lightest reported deployment occupies on the order of 80 kB of RAM [23] against the 8 kB of SRAM of the target device.

### 2.3. From Spatial to Temporal Reference: The Cell Distribution Assumption

In the IoT mesh sensor application, no spatial reference is available. Each node produces a single scalar time series *x*[*n*] sampled at f_s_ = 100 Hz. The only stationarity available is temporal: the current frame resembles recent frames if no event is present. All five detectors in this study use a temporal reference window in place of the spatial reference cells of canonical radar CFAR. For CA-CFAR and OS-CFAR, the reference cells are the most recent *N* frame statistics from the same node, with one guard frame preceding the current frame. For TSNFA, the same principle applies, but it is per bin in the frequency domain. For CUSUM, the reference is the running minimum or maximum of the cumulative log-likelihood-ratio score. For Lipski FFT, the reference is a per-bin (mu_b, sigma_b) pair estimated during calibration and slow-tracked via EMA on no-detection frames.

A deeper assumption of the textbook CFAR derivation is that each cell is exponentially distributed. In the classical radar setting, a cell is the squared magnitude |I|^2^ + |Q|^2^ of a single complex Fourier-domain sample, where I and Q are independent Gaussian noise components; their squared sum is chi-squared with two degrees of freedom, which is exactly exponential. This distribution is what the threshold calibration is built on: a false alarm is the event that a noise-only cell exceeds α·Z, so the multiplier is obtained by solving P(cell > α·Z) = P_fa_, and the exponential tail makes this solvable in closed form, yielding the Finn-Johnson expression of Equation (3) and the Rohling form used by OS-CFAR. For a long reference window, Z approaches the true mean and α approaches ln(1/P_fa_), which at P_fa_ = 10^−3^ is 6.91; this asymptotic value reappears when the TSNFA threshold is discussed (Section 4.1).

In the IoT mesh sensor setting, the natural frame statistic for CA-CFAR and OS-CFAR is the sum of squared sample magnitudes *X*(*m*) = Sum|*x*[*n*]|^2^ over the 128 samples of a frame. By the central limit theorem, this is approximately Gaussian with mean L·P (=128*P*) and standard deviation sqrt(2*L*)·*P* (=16*P*). The cell distribution is therefore Gaussian-bell-shaped, not exponential. The threshold “*a* · *Z*”, derived from the exponential calibration, lands approximately 54 standard deviations to the right of the bell’s centre, in a region pure thermal noise effectively never reaches. The detector therefore relies on heavy-tailed structured interferes (mains harmonics, digital bursts, drift excursions) to cross the threshold, and the empirical false alarm rate is governed by the statistics of those interferers rather than by the calibrated P_fa_. The CFAR algorithm is not at fault; the frame-statistic choice forced by the single-node hardware constraint takes the detector outside the regime its calibration was designed for.

TSNFA addresses this constraint at the FFT step rather than at the threshold. The per-bin statistic |*X_k_*|^2^ = Re(*X_k_*)^2^ + Im(*X_k_*)^2^ is the squared magnitude of a single complex Fourier coefficient. The real and imaginary parts are each approximately Gaussian (a linear combination of 128 input Gaussians at fixed FFT weights), and their squared sum is chi-squared with two degrees of freedom exponential. The cell distribution assumption of the textbook CFAR derivation is therefore restored, per bin, by the FFT itself. This is an exact algebraic property of the FFT basis under wide-sense stationary input. The per-bin threshold multiplier ζ = 6.0 used by the unconfirmed TSNFA variants sits close to the asymptotic exponential tail value ln(1/P_fa_) = 6.91 at P_fa_ = 10^−3^, though it was derived from an empirically found value from deployed hardware. The convergence is not a coincidence: both detectors implicitly target the same per-cell tail probability on the same exponential distribution, where TSNFA arrives via the FFT and CA-CFAR does not arrive at it at all in our setting. The recommended configuration operates at ζ = 2.5 with three-frame confirmation (Section 4.1); the single-frame tail argument then applies to the raw trigger, and the confirmation rule supplies the remaining false alarm suppression as binary integration [13]. This restored per-bin exponential statistic is the central methodological claim of the present work: the FFT step does not merely filter interference, it re-establishes the distributional assumption on which CFAR calibration rests.

Every detector in this study is run at its canonical published parameters; we do not tune comparators to the simulation environment. If a published detector is re-tuned for the comparison environment, the comparison no longer measures the published detector. Canonical settings are: CA-CFAR P_fa_ = 10^−3^, OS-CFAR P_fa_ = 10^−3^ with k = 3N/4 [4], CUSUM a_fa_ = 10^−5^ after Torre et al. [15]. The Lipski comparator is the one exception: its published multiplier (k = 0.1) is tied to a broadband moving average statistic and does not transpose to per-bin spectral magnitudes, so the adaptation runs at the untuned three-sigma default k = 3 (Section 4.2).

## 3. Materials and Methods

All five detectors are evaluated against a common synthetic signal generated for each frame of each Monte Carlo run on a simulated mesh network. This section describes the signal model, the network topology and medium access, the configuration matrix, and the reported metrics. The complete simulator source code, configuration files, and per-frame trigger-strength traces from which all reported results are computed are provided as Supplementary Materials (Zenodo [32]).

### 3.1. Common Signal Model

The signal at sample index *n* within frame m for node i is given by Equation (1):

x_i_[*n*] = s[*n* − τ_i_] + wth[*n*] + w_EMI_[*n*] + w_dig_[*n*] (1)

where:

- s[*n* − τ_i_] is the event waveform, a damped sinusoid in the [1,5] Hz band, injected at the SNR level of the test configuration (Section 3.3) relative to the in-band noise power P, with per-node onset delay τ_i_;
- wth[*n*] is zero-mean white Gaussian thermal noise;
- w_EMI_[*n*] is 50 Hz mains interference at amplitude 0.3 sqrt(P);
- w_dig_[*n*] represents intermittent digital switching bursts at 800 to 2000 Hz with amplitude up to 2.0·sqrt(P);
- P is the in-band noise power, which drifts sinusoidally over a one-hour cycle with ±6 dB excursion, so that several full drift cycles occur within each 24 h run.

### 3.2. Network Topology and Mesh

For 10 nodes, the area is 350 m × 350 m; for 50 nodes, 750 m × 750 m. Each node has 200 m radio range. Mesh routing is multi-hop shortest path to the sink. Medium access is CSMA/CA with CW_min = 8, CW_max = 64, slot 320 us, four retries, reflecting LoRa-class radio assumed for the deployed hardware.

### 3.3. Configuration Matrix

Events are scheduled per node by an independent Poisson process with rate λ = 1.0 event per node per hour. Event waveforms span 5 s (3.9 frames at the 1.28 s frame period). The duration is hardcoded rather than drawn from a distribution: 5 s is chosen as representative of the target event class (human movement at a perimeter boundary) and exceeds the 3.84 s persistence minimum imposed by the γ_d_ = 3 trigger filter (Section 4.1). Holding the duration constant isolates signal margin as the only variable along the SNR frontier; we refer to it below as the chosen event duration. All detectors process the same realisation at each Monte Carlo seed; trigger outputs are cross-referenced against ground-truth events.

The campaign is organised as an SNR frontier at fixed network size, plus scale checks. Nine SNR levels (1.5, 2, 3, 4, 5, 6, 12, 18, and 24 dB) are simulated at 10 nodes; the 3, 12, and 18 dB levels are repeated at 50 nodes; and one additional configuration (10 nodes, 12 dB) measures four further members of the classical CFAR family (GO-CFAR, SO-CFAR, TM-CFAR, VI-CFAR; the tier 2 detectors of Section 2.2), referred to below as the CFAR-family configuration. Each test configuration is replicated five times over 24 h of simulated operation. The two network sizes are not a density sweep: they represent the qualitative regimes of a sparse deployment (10 nodes, short relay paths, low channel contention) and a dense deployment (50 nodes, multi-hop relay load, contention at the sink), and the scale checks test whether per-node detection statistics survive the transition between the two regimes. The false-positive relay load grows with node count and is therefore bounded by the 50-node test configurations; we assume that intermediate sizes between 10 and 50 nodes interpolate between the two regimes without introducing a distinct failure mechanism. The SNR range is anchored at both ends: 24 dB represents a strong nearby event, 12 to 18 dB is the design regime of the deployed system, and the 1.5-to-6 dB band brackets the detection frontier, where detection rates of the tested detectors transition from near zero to near one. The lower bound of 1.5 dB lies below the 50% detection point of the best-performing detector, so the frontier is bracketed rather than truncated. The configuration matrix is summarized in Table 1.

### 3.4. Reported Metrics

Five metrics are reported per detector per configuration: event detection rate (fraction of scheduled events with at least one trigger within the event window); event precision (fraction of detected events with at least one true-positive and zero false-positive triggers); false-positive cluster count (false-positive triggers are counted as one single false positive count unless separated by at least a 5 s gap); per-node network load (mean bytes per hour transmitted by each node); and mean detection latency (time for event onset to first true-positive trigger at the sink).

### 3.5. Embedded Implementation and Resource Measurement

**Figure 1:**
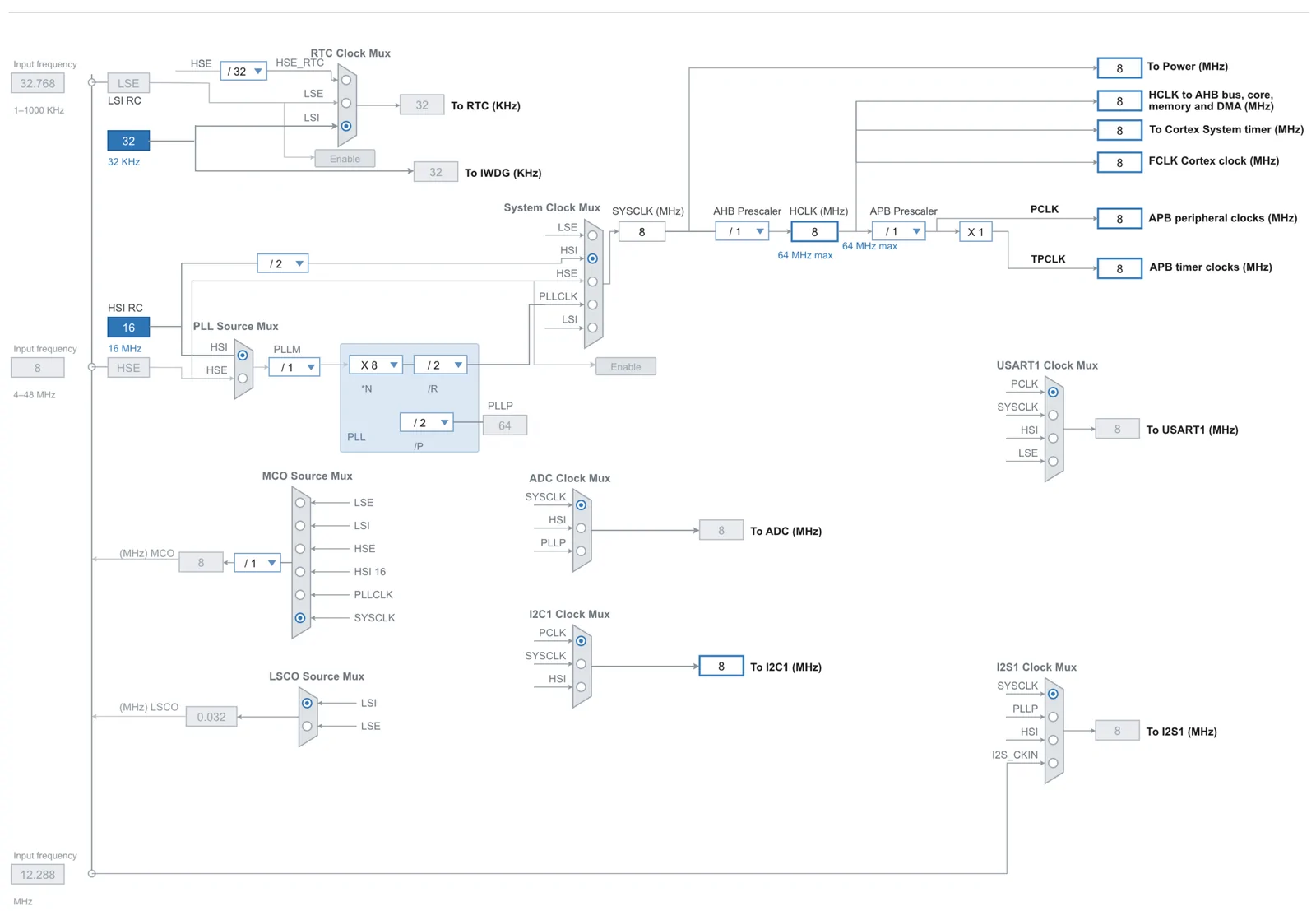
STM32CubeIDE clock configuration: the 16 MHz HSI RC oscillator is divided to SYSCLK = 8 MHz, with HCLK = PCLK = ADC_CLK = 8 MHz; the RTC and independent watchdog run from the 32 kHz LSI; HSE and LSE are unpopulated. Trapezoids are clock multiplexers (filled radio button = selected input), dropdown boxes show prescaler settings, blue marks the active oscillators and derived clocks, and grey marks unused features (PLL, MCO, LSCO).

The deployed node is an STM32G030F6 (Arm Cortex-M0+, 32 kB Flash, 8 kB SRAM, no FPU, no hardware divide). Clocking is fully internal: the 16 MHz HSI RC oscillator feeds a divide-by-two to give SYSCLK = HCLK = PCLK = ADC_CLK = 8 MHz, the RTC and the independent watchdog run from the 32 kHz LSI, and no external crystal is populated (Figure 1). At 8 MHz, the Flash operates with zero wait states. The 1% HSI frequency tolerance shifts bin centres by at most 0.008 Hz at the 0.78 Hz bin width, two orders of magnitude below the bin width and immaterial for detection. Frames are 128 samples at fs = 100 Hz (Tb = 1.28 s). Measured core current with the detector running is 3.2 mA at 3.0 V.

The spectral stage is implemented as a 128-point radix-2 Cooley-Tukey FFT [33] in Q15 format with per-stage right-shift scaling and block floating point. Without recovery of the block exponent, fixed scaling destroys the small in-band magnitudes on which the noise-floor estimate depends: the mean bin-magnitude error reaches 85% and falls to 0.21% with block floating point at the cost of one maximum-search pass and 12 multiplies per frame. Fidelity of the fixed-point front end was verified over 281,250 frames (100 h of simulated time under the signal model of Section 3.1) against an exact double-precision DFT, giving a mean relative bin-magnitude error of 0.21% and a cosine similarity of 0.999999. This verification concerns the FFT front end only; detector-level performance is reported in Section 5. The measured FFT cost is 1804 multiplies, 3718 additions, and 5452 memory accesses per frame, approximately 2.2 × 10^4^ cycles or 2.77 ms at 8 MHz, which is 0.22% of the frame budget; the 128-point FFT is therefore not a bottleneck at 8 MHz, with more than two orders of magnitude of margin. A six-filter Goertzel bank is an equivalent option: for integer bin indices, its outputs equal the corresponding DFT coefficients, so the per-bin distribution argument of Section 2.3 is preserved, while Flash and SRAM fall by approximately 0.9 kB and 512 B, respectively.

Table 2 reports the per-detector resource increments over the base node firmware. Flash increments for CA-CFAR, OS-CFAR, and CUSUM are analytical estimates from code volume; all other figures come from the implemented code. Cycle counts per frame are 8.1 × 10^4^ (TSNFA with FFT), 6.7 × 10^4^ (Goertzel bank), 1.4 × 10^4^ (Goertzel with incremental median), 2.3 × 10^4^ (Lipski FFT), 1.0 × 10^3^ (CA-CFAR), 2.6 × 10^3^ (OS-CFAR), and 1.2 × 10^3^ (CUSUM), derived from operation counters in an instrumented build under an ARMv6-M timing model (single-cycle multiply, add, subtract, and shift; two-cycle load and store; four cycles per compare-and-move pair; plus 15% loop control). MCU energy assumes the measured 3.2 mA at 3.0 V and 2812 frames per hour.

Four properties of the ARMv6-M architecture shaped the implementation. First, there is no 64-bit multiply, so every product must fit in 32 bits; in the FFT, this is guaranteed by the unconditional per-stage right shift (overflow is impossible and the worst-case execution time is constant), and in the Goertzel bank the input is scaled by three bits, bounding the state to 16,384 and every product below 2^31^. Second, there is no hardware divide and no square root, so the detector operates in the squared-magnitude domain: the trigger compares the squared bin magnitude against the squared threshold using a saturating multiply, with the squared threshold coefficient precomputed at calibration. The median commutes with monotone transforms, so the median of squares equals the square of the median, and the equivalence with the reference formulation is exact. Third, worst-case execution time is deterministic: all of the state is statically allocated, with no heap and no recursion. The 64-entry median cascade dominates the frame budget at 7.33 of 10.09 ms; incremental insertion into a sorted array reduces the frame time to 1.71 ms at a cost of 1536 B of additional SRAM, and at 0.79%, CPU load plain insertion sort (worst case 2016 comparisons per bin) is retained as the baseline. Fourth, the Cortex-M0+ has no debug cycle counter, so execution time is measured with a hardware timer, and the cycle figures above are derived from instruction counters in an instrumented build. The noise-floor estimate requires 64 frames (81.9 s) of warm-up after power-on, during which the trigger is inhibited.

**Figure 2:**
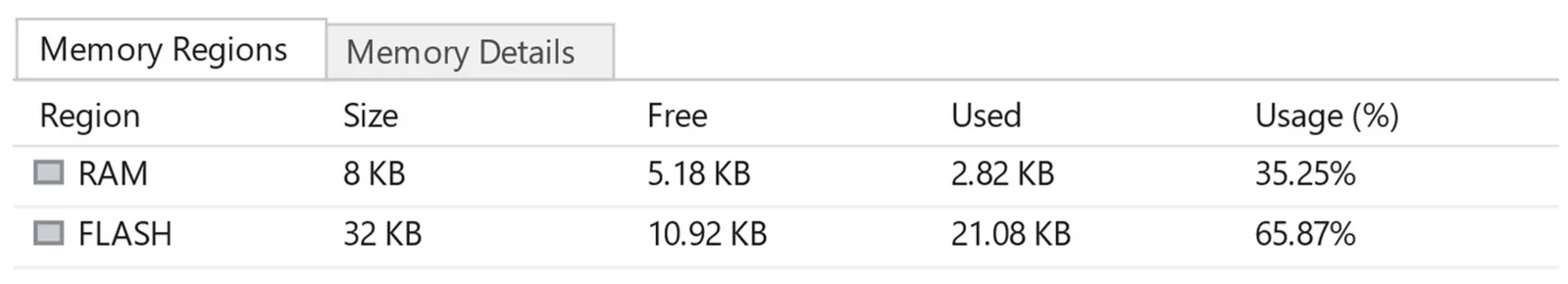
STM32CubeIDE memory-usage report for the Release build of the node firmware.

**Figure 3:**
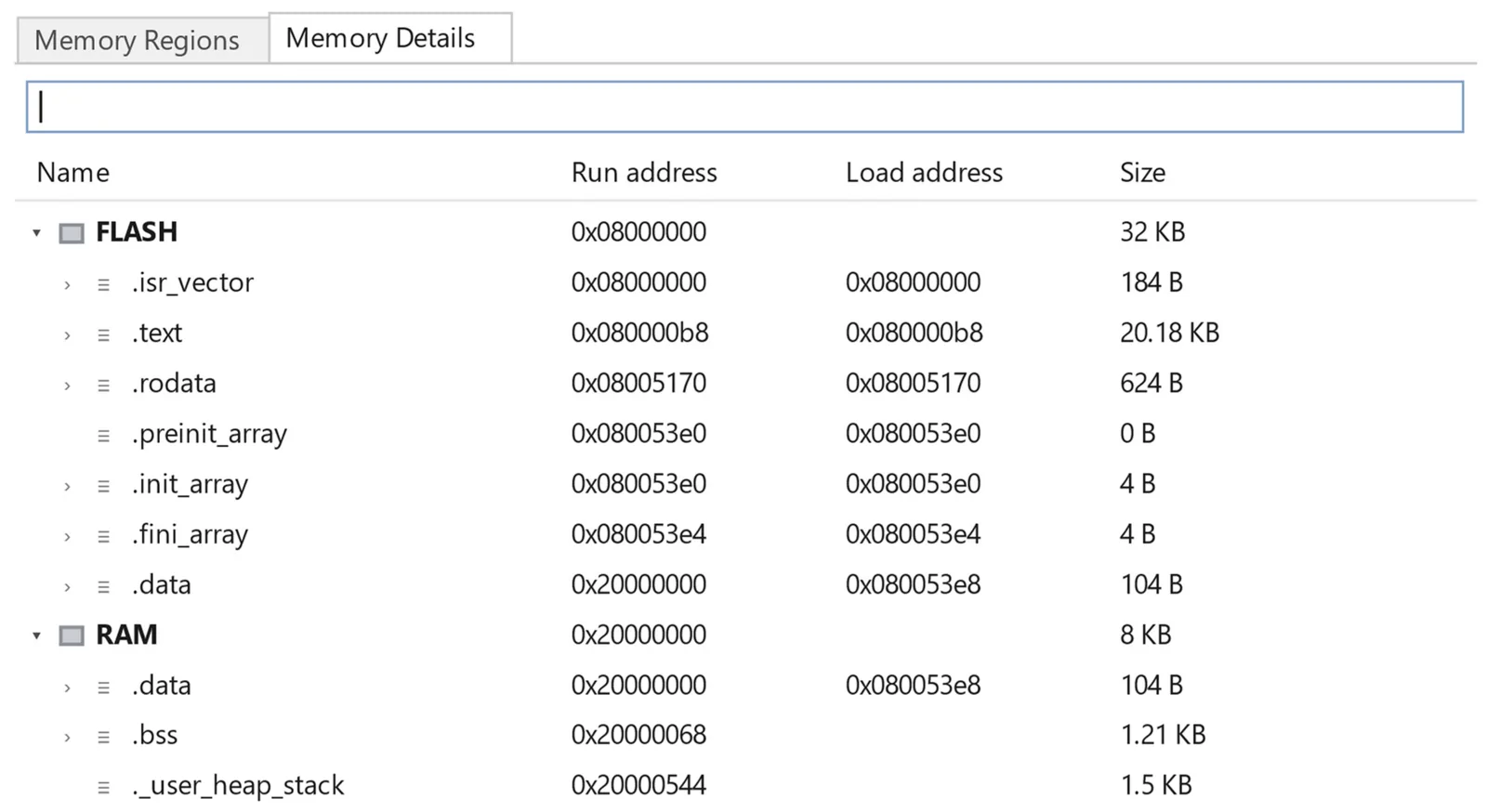
Section-level memory breakdown for the same Release build.

Linker measurements (STM32CubeIDE, Release build; Figure 2 and Figure 3) place the base node firmware without the spectral stage at 21.08 kB of Flash (65.9%) and 2.82 kB of SRAM (35.3%), of which 1.5 kB is reserved for heap and stack. With the full TSNFA cascade on the 128-point FFT, the totals become approximately 22.8 kB of Flash (71.2%) and 5.20 kB of SRAM (65.0%); with the Goertzel bank, 21.9 kB (68.4%) and 4.69 kB (58.6%). Both variants fit the STM32G030F6 budget without moving to a larger device.

## 4. Detection Algorithms

**Figure 4:**
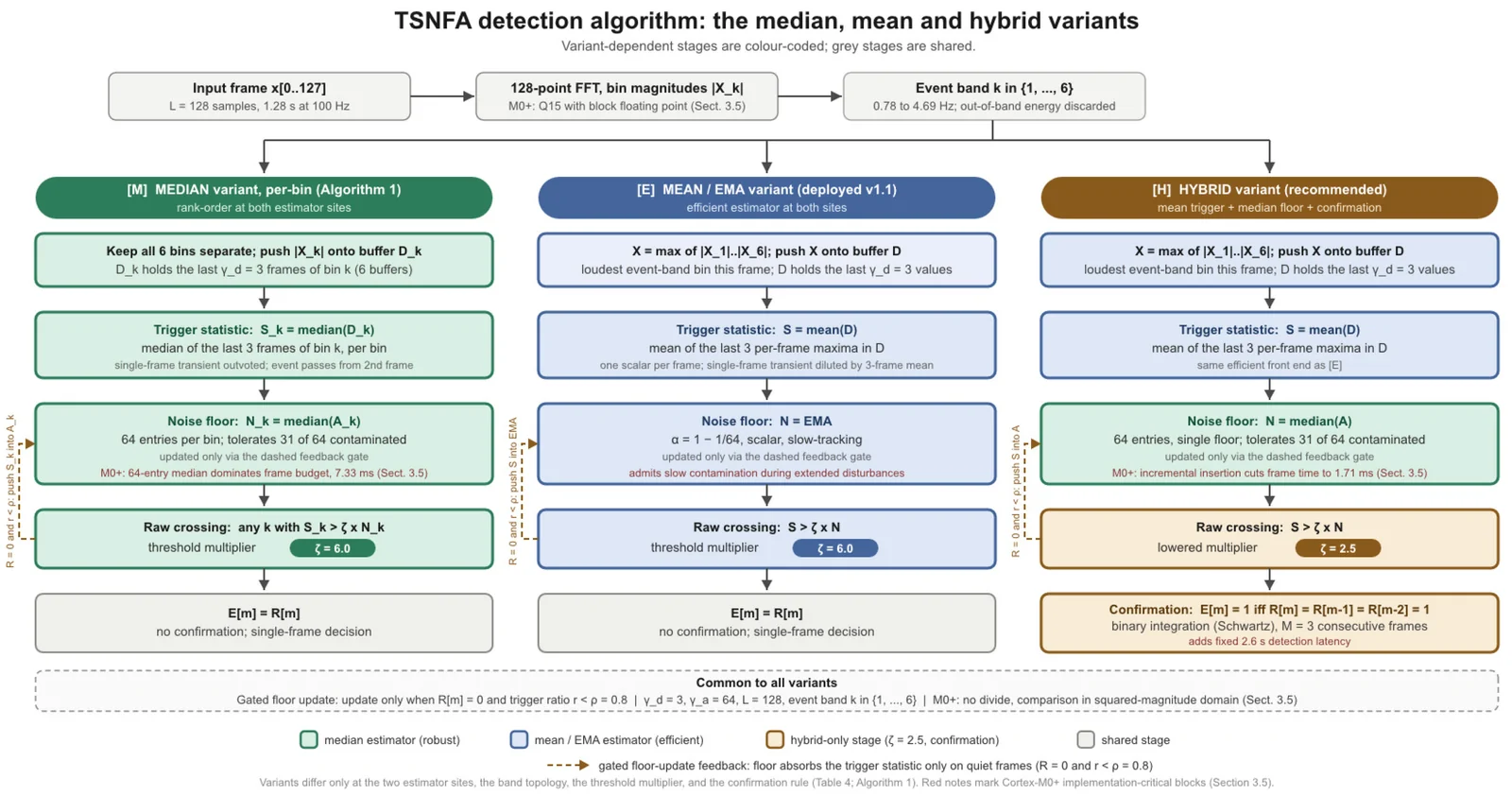
Algorithm flow diagram for TSNFA [12]. The three variants share the front end and differ only downstream. Colour fills identify the estimator family: green for the robust median, blue for the efficient mean/EMA, amber for the hybrid-only stages (ζ = 2.5, three-frame confirmation); grey stages are shared. Red notes mark Cortex-M0+ implementation-critical blocks (Section 3.5); the dashed box collects common parameters.

**Figure 5:**
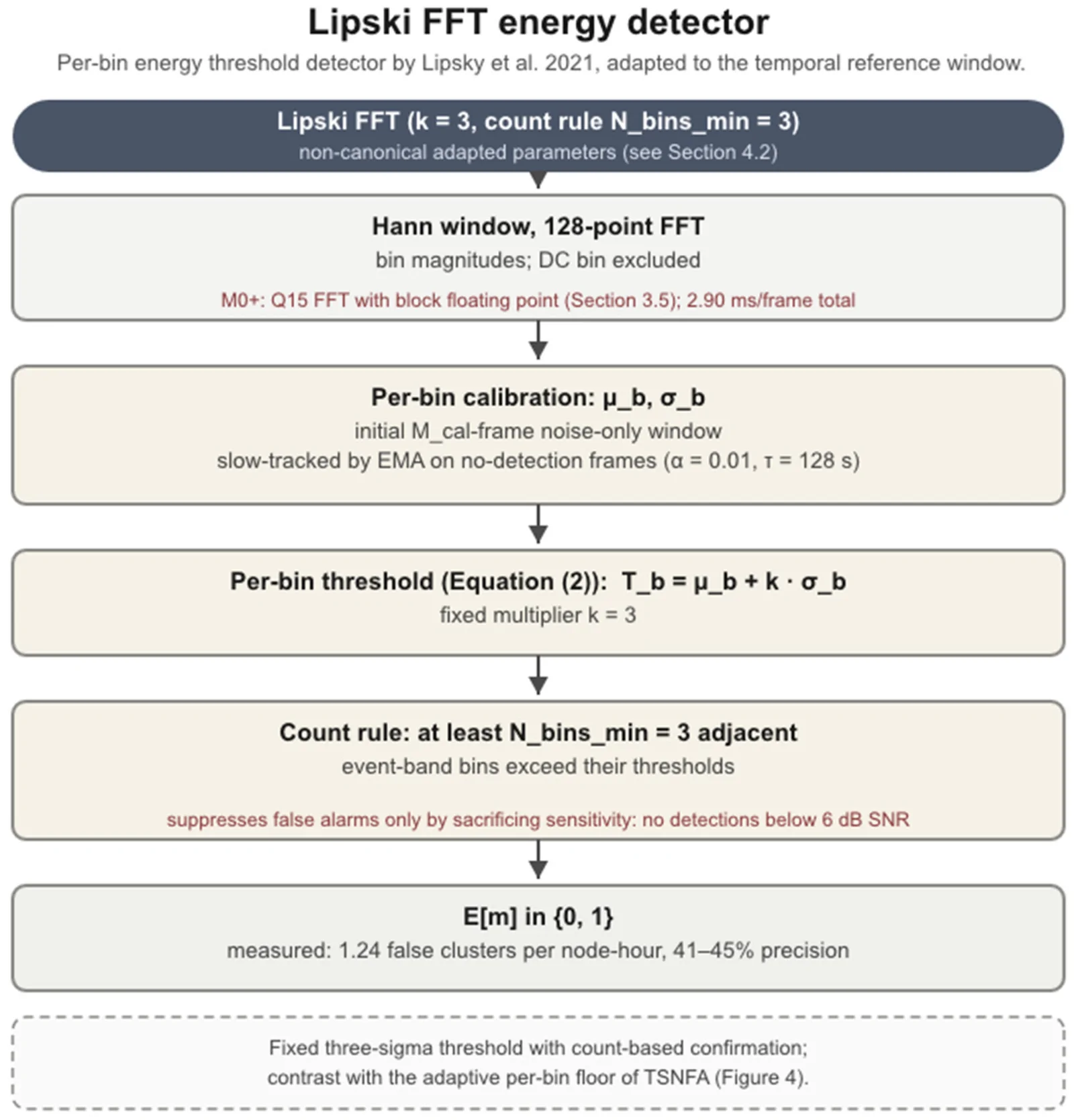
Algorithm flow diagram for the per-bin FFT adaptation of the Lipski energy detector [29]. Color coding: Light brown stages are specific to the Lipsky detector; grey stages are the frame-statistic input and trigger output shared with the other broadband detectors; red notes mark Cortex-M0+ implementation details (Section 3.5); the dashed box states the detector’s characteristic failure mode.

**Figure 6:**
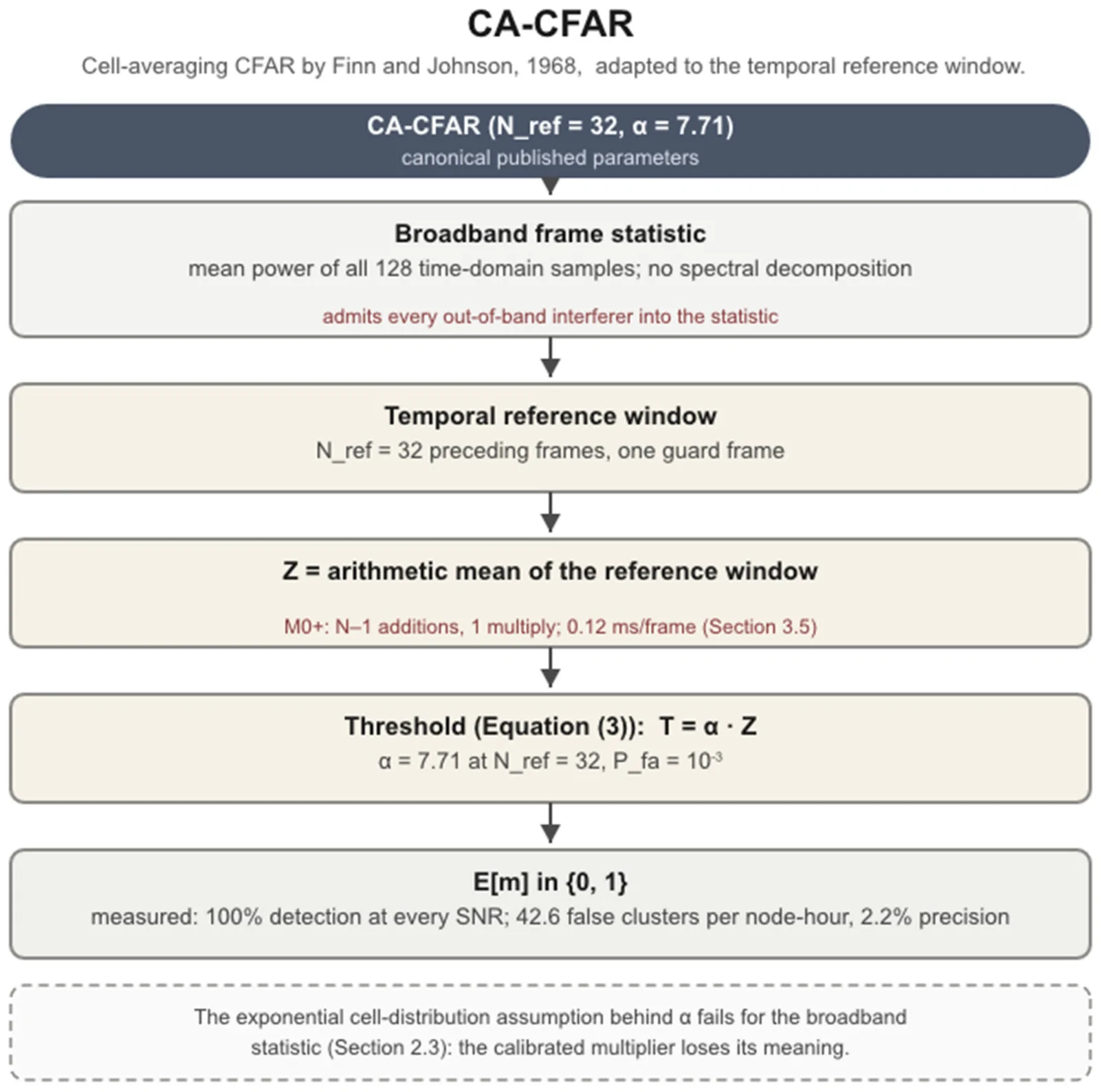
Algorithm flow diagram for CA-CFAR [3] adapted to the temporal reference window. Color coding: Light brown stages are specific to the CA-CFAR detector; grey stages are the frame-statistic input and trigger output shared with the other broadband detectors; red notes mark Cortex-M0+ implementation details (Section 3.5); the dashed box states the detector’s characteristic failure mode.

**Figure 7:**
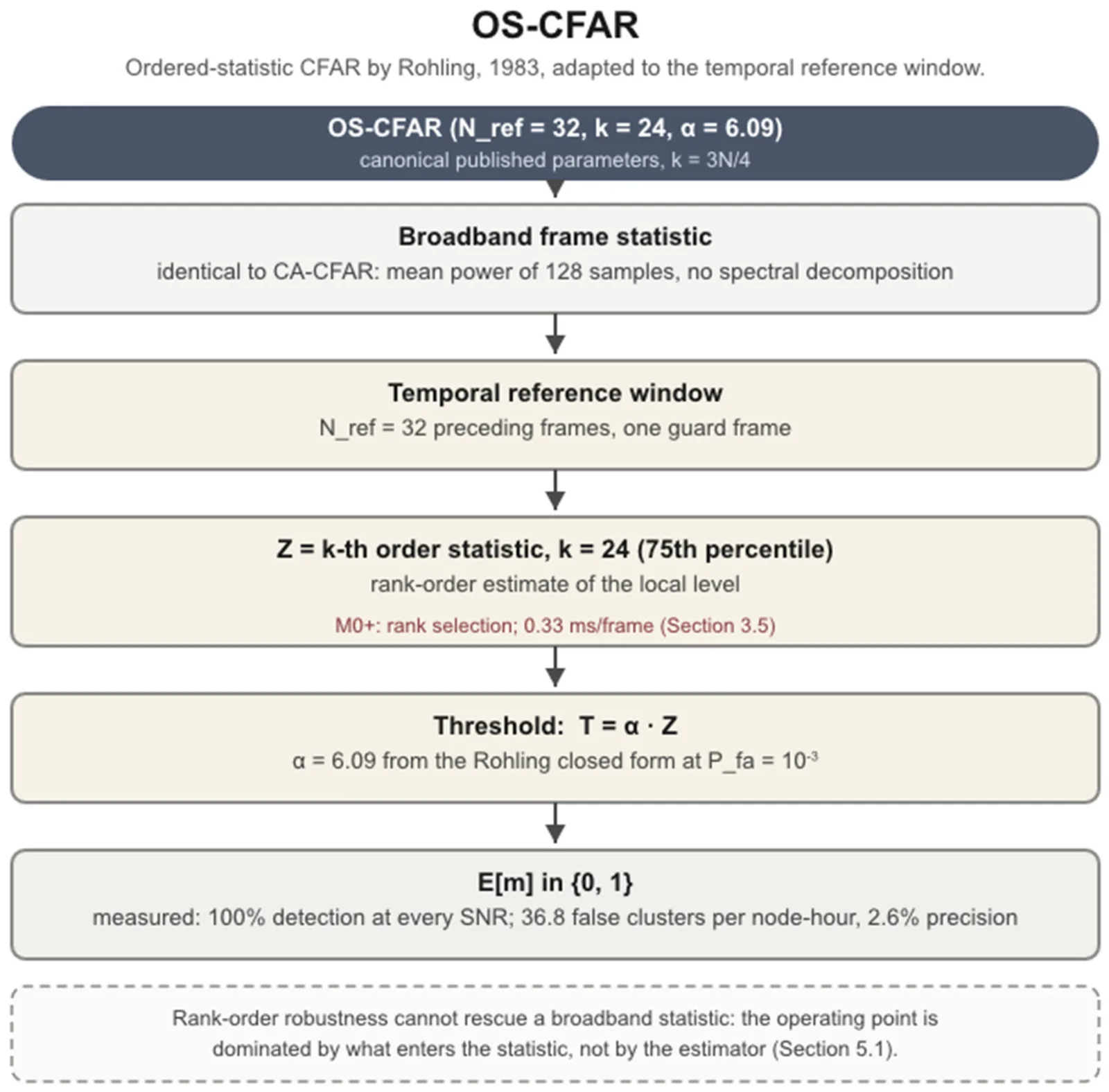
Algorithm flow diagram for OS-CFAR [4] adapted to the temporal reference window. Color coding: Light brown stages are specific to the OS-CFAR detector; grey stages are the frame-statistic input and trigger output shared with the other broadband detectors; red notes mark Cortex-M0+ implementation details (Section 3.5); the dashed box states the detector’s characteristic failure mode.

**Figure 8:**
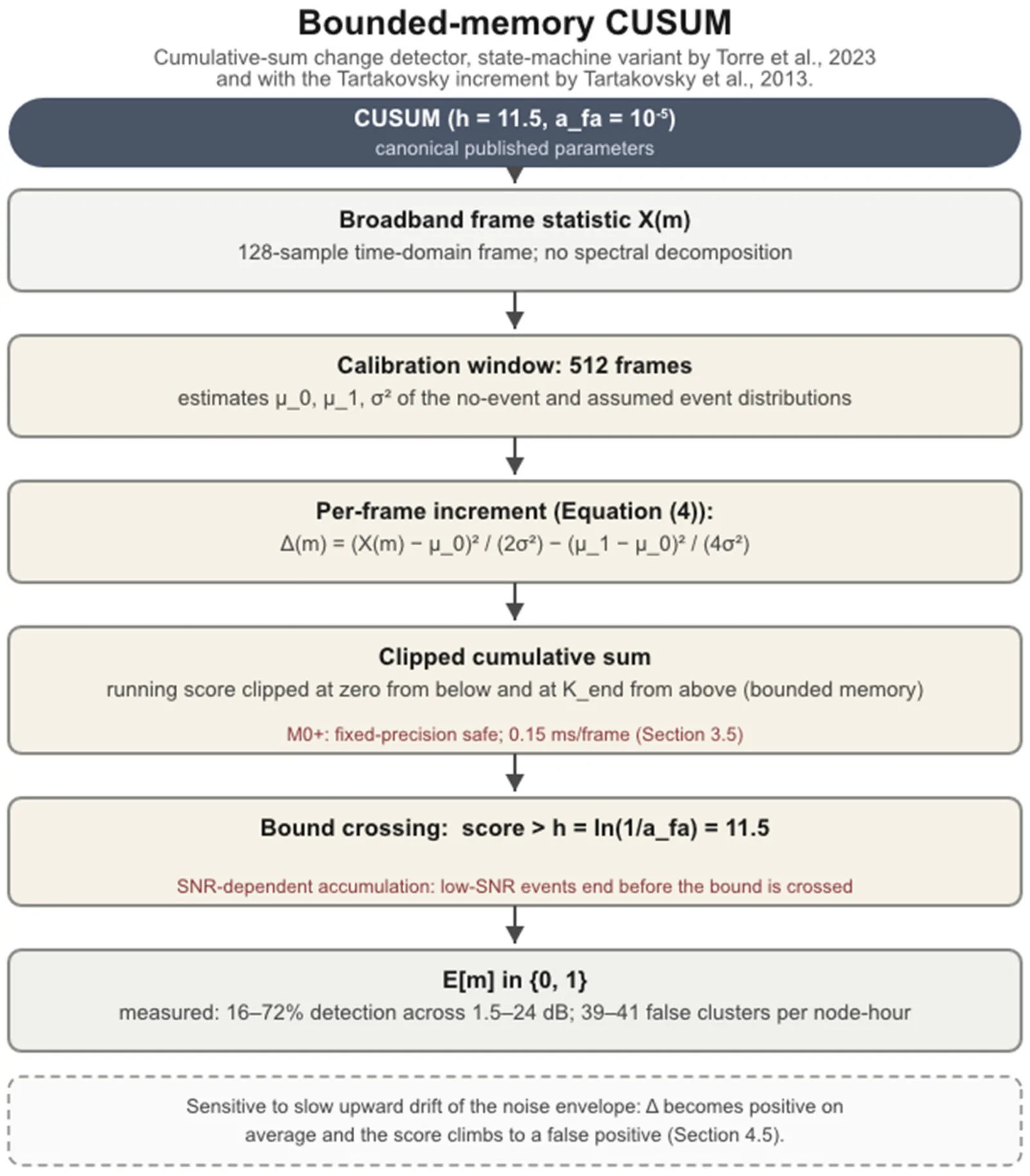
Algorithm flow diagram for the bounded-memory CUSUM variant [15,16]. Color coding: Light brown stages are specific to the CUSUM detector; grey stages are the frame-statistic input and trigger output shared with the other broadband detectors; red notes mark Cortex-M0+ implementation details (Section 3.5); the dashed box states the detector’s characteristic failure mode.

The five detectors are presented in order of architectural complexity: TSNFA first (the proposed cascade), then the four classical comparators. For each algorithm, we provide an overview and a parameter table. The TSNFA pseudocode is given in full because it is the proposed detector (Algorithm 1); compact pseudocode for all comparators, including the tier 2 CFAR-family detectors, is collected in Algorithm 2 at the end of this section. A flow diagram is provided per algorithm in Figure 4, Figure 5, Figure 6, Figure 7 and Figure 8, each annotating the blocks that raised implementation difficulty on the Cortex-M0+ (Section 3.5). Reference implementations of all five are available on the data page.
**Algorithm 1.** TSNFA frame processing. Variant-dependent lines are tagged [M] median (per-bin, rank-order), [E] mean/EMA (deployed v1.1), and [H] hybrid (recommended)Input: frame x[0..L-1]; per-bin buffers for [M] are D_k_, A_k_; scalar buffers for [H] are D, A; scalar buffer for [E] is N; Params: γ_d_ = 3, γ_a_ = 64, ζ (Table 3), ρ = 0.8, M = 3 [H] Output: trigger E[m]; noise floor(s)X_k_ <- |FFT(x)_k_| for k in K = {1,…,6} // bin magnitudes[M] for each k: push X_k_ onto D_k_; S_k_ <- median(D_k_)[E,H] X <- max over k of X_k_; push X onto D; S <- mean(D)[M] N_k_ <- median(A_k_) for each k // per-bin floors[E] N <- EMA state (α = 1 − 1/γ_a_) // single floor[H] N <- median(A) // single floor[M] r <- max over k of S_k_/(ζ x N_k_)[E,H] r <- S/(ζ x N)R[m] <- 1 if r > 1 else 0 // raw crossingif R[m] = 0 and r < rho then // gated floor update[M] for each k: push S_k_ onto A_k_[E] N <- α x N + (1 − α) x S[H] push S onto A[M,E] E[m] <- R[m][H] E[m] <- 1 if R[m] = R[m-1] = … = R[m-M+1] = 1 else 0 // confirmationreturn E[m], noise floor(s)

**Algorithm 2.** Comparator frame processing, compact form. The broadband detectors share the frame statistic *X*(*m*) and the temporal reference window (N_ref_ = 32 frames, one guard frame); the tier 2 CFAR-family detectors differ from CA-CFAR only in the estimator line Z (Section 2.2)Shared:X(m) <- sum over n of |x[n]|^2^               // broadband frame statistic window <- last N_ref_ = 32 frame statistics, one guard frameCA-CFAR:Z <- mean(window);               E[m] <- 1 if X(m) > 7.71 x ZOS-CFAR:Z <- 24th order statistic;      E[m] <- 1 if X(m) > 6.09 x ZTier 2 substitutions (same skeleton, Z line only):GO-CFAR:Z <- max( mean(leading half), mean(lagging half) )SO-CFAR:Z <- min( mean(leading half), mean(lagging half) )TM-CFAR:Z <- trimmed mean of the sorted windowVI-CFAR:Z <- CA, GO, or SO logic, selected by variability index and mean ratio (CMLD, GCMLD, and RVI-CFAR are further Z substitutions, cited but not simulated; Section 2.2)Lipski:Hann window, FFT, DC bin excluded per bin b: R_b_ <- 1 if |X_b_| > µ_b_ + 3 x σ_b_        // Equation (2) E[m] <- 1 if R_b_ >= 3 adjacent event-band bins fire if E[m] = 0: EMA update of (µ_b_, σ_b_)CUSUM:S <- clip(S + Δ(m), 0, K_end_)             // Delta from Equation (4) E[m] <- 1 if S > h = 11.5

### 4.1. TSNFA: Variants and Recommended Configuration

TSNFA combines interlocking defences in a fixed order: spectral band selection, then a trigger statistic, then adaptive noise-floor tracking, then temporal confirmation. Two of these stages are estimator sites, and the choice of estimator at each site defines a variant. The trigger site computes the statistic that is compared against the threshold; the floor site computes the noise-floor estimate that scales the threshold. Three variants are evaluated in this study: the “*median variant*”, the per-bin rank-order formulation of the TSNFA algorithm, which uses the median at both sites; the “*mean variant*”, which reproduces the deployed v1.1 detector with a short-window mean at the trigger site and an exponential moving average (EMA) at the floor site; and the “*hybrid variant*”, which keeps the mean-trigger front end and replaces only the floor estimator with the median, adding a confirmation rule. The hybrid variant is the recommended configuration. It is an empirical finding of the present study, selected by the estimator ablation of Section 5.2 rather than taken from the original specification [12]. The median variant as simulated carries two corrections relative to the original specification [12], validated in the verification study distributed with the archived simulator (simulator_v1_2.py [32]): processing is fully per bin, with each event-band bin holding its own buffers and noise floor, and the trigger compares the γ_d_-frame median rather than the instantaneous bin magnitude, so that Defence 2 acts in the trigger path.

Defence 1, spectral band selection: the 128-point FFT decomposes each frame into 64 bins. Only the six bins in K = {1, …, 6}, covering 0.78 to 4.69 Hz at 0.781 Hz resolution, are retained; all energy from out-of-band sources is discarded before any detection statistic is computed. The reference formulation operates on the bin magnitudes |X_k_|. The microcontroller has no square-root instruction, so the embedded implementation compares squared values instead: |*X_k_*|^2^ against *ζ*^2^ × N^2^, with *ζ*^2^ precomputed at calibration. Because squaring does not change which of two values is larger, both versions reach the same trigger decision on every frame (Section 3.5). Defence 2, the trigger statistic (γ_d_): the median variant maintains a γ_d_ buffer per bin and uses the buffer median as the trigger statistic, so a single-frame in-band transient (an ADC glitch, a wind gust) is outvoted, while a persistent event passes from its second frame. The mean and hybrid variants first take the maximum across the event-band bins and use the γ_d_ -frame mean of that maximum as a single scalar trigger statistic, matching the deployed v1.1 front end. With γ_d_ = 3, both filters reject single-frame transients, while an event of the chosen 5 s duration (Section 3.3), spanning 3.9 frames, persists at full magnitude.

Defence 3, the noise floor (γ_a_): the median and hybrid variants use the median of a γ_a_ buffer, whose breakdown point floor, ((γ_a_ − 1)/2) = 31 entries, makes the estimate insensitive to contaminated entries; the mean variant slow-tracks with an EMA at coefficient α = 1 − 1/ γ_a_. In all three variants, the floor update is gated: it is performed only on frames with no raw threshold crossing and with the trigger ratio below a hysteresis margin ρ = 0.8, so event energy and near-threshold excursions cannot inflate the floor. The median floor adds robustness to any contamination that passes the gate.

Defence 4, confirmation: the hybrid variant declares an event only after M = 3 consecutive raw trigger frames. This is binary integration in the sense of Schwartz [13]: “uncorrelated threshold excursions” must coincide on three consecutive frames to produce a false positive, while an event of the chosen duration (3.9 frames, Section 3.3) survives. (For “uncorrelated threshold excursions”, in our case, the events are correlated with a high likelihood correlated. Therefore, we divert from Schwartz [13] in this aspect. That is why we refer to the implementation as a “sense of Schwartz”.) Confirmation permits a lower threshold multiplier (ζ = 2.5 against 6.0 for the unconfirmed variants), which is what recovers low-SNR sensitivity. The cost is deterministic: confirmation adds two frame periods (2.6 s) of detection latency, which is not captured by the network latency metric of Section 3.4 and is stated separately wherever latency is reported.

For operation without the confirmation rule, the deployed setting ζ = 6.0 was set empirically from field hardware, before any of the present analysis, and lands within 15% of the asymptotic exponential-tail value 6.91 at P_fa_ = 10^−3^ (Section 2.3).

The recommended operating point, ζ = 2.5 with M = 3, is a new finding of this study and is not part of the original specification [12]. It was identified from the per-threshold analysis of Section 5.5: at ζ = 6.0 without confirmation, the mean and median variants miss the low-SNR events whose per-frame magnitudes cannot reach 6.0·N; the hybrid variant lowers the per-frame threshold to 2.5, which those events can reach, and its three-frame coincidence requirement supplies the false alarm suppression the lower threshold gives up (for comparisons between the algorithms see Section 5.1).

The median variant preserves the per-bin structure of the original specification, with the OR across bins expressed in line 4 as the maximum of the per-bin ratios, which is algebraically identical. The mean and hybrid variants collapse the band to its maximum bin before filtering, matching the deployed v1.1 front end. The variants therefore differ only in lines 2, 3, 6, and 7 and in the value of ζ; Table 4 summarises the mapping. The gated floor update of line 6 is common to all variants. The embedded implementation performs the comparison of line 4 in the squared-magnitude domain, which is exact for the median stages because the median commutes with monotone transforms (Section 3.5).

### 4.2. Lipski FFT Energy Detector (k = 3)

The Lipski comparator is a per-bin FFT adaptation of the adaptive-threshold energy detector of Lipski et al. [29], transposed to the frame structure of Section 3.1 in the same way as CA-CFAR and OS-CFAR. The Lipski et al. threshold is a broadband moving-average energy statistic at µ + k·σ and reports k = 0.1 as its operating value; on that statistic, the standard deviation is small against the mean, so k is small. Transposed to per-bin spectral magnitudes, where σ_b_ is comparable to µ_b_, the same rule requires a multiplier of order unity, and the published value does not transpose. We adopt k = 3, the standard three-sigma outlier criterion, as the untuned default for a per-bin statistic; this was the deployed v1.1 setting, fixed before the present comparison, and the threshold sweep of Section 5.3 confirms that it sits at the favourable end of the comparator’s own trade-off. In our implementation, a Hann window is applied to the time-domain samples before the FFT, and the DC bin is excluded. The per-bin detection threshold is given in Equation (2):

T_b_ = μ_b_ + k·σ_b_ (2)

where:

- T_b_ is the detection threshold of bin b;
- µ_b_ is the per-bin mean, calibrated during an initial M_cal_-frame noise-only window and slow-tracked via an EMA on no-detection frames (α = 0.01, τ = 128 s);
- σ_b_ is the per-bin standard deviation, calibrated and tracked identically;
- k is the threshold multiplier, at the three-sigma default k = 3 adopted above.

The detector fires when at least N_bins_min_-adjacent bins in the event band exceed their thresholds (N_bins_min_ = 3, the count rule of the reference implementation). The Lipski parameters are listed in Table 5.

### 4.3. CA-CFAR (Cell-Averaging)

CA-CFAR [3] computes the threshold from the arithmetic mean of N_ref_ reference cells. Adapted to single-node operation, the reference cells are the most recent N_ref_ frame statistics from the same node, with one guard frame between the reference window and the current frame. The frame statistic is the broadband energy proxy *X*(*m*) = Sum|*x*[*n*]|^2^. The threshold multiplier follows the Finn-Johnson formula given in Equation (3):

α = N_ref_ × (P_fa_^(−1/Nref)^ − 1) (3)

where:

- N_ref_ is the number of reference cells (frames) in the sliding window;
- P_fa_ is the target false alarm probability per decision;
- α is the resulting threshold multiplier applied to the reference mean.
- At N_ref_ = 32 and P_fa_ = 10^−3^, this gives α = 7.71. CA-CFAR is exposed to the broadband noise environment because the frame statistic does not perform spectral filtering.

### 4.4. OS-CFAR (Ordered Statistic)

OS-CFAR [4] replaces the cell-averaging mean with the k-th order statistic of the reference window. Following Rohling [4], k = 3N/4. With N_ref_ = 32, this gives k = 24 (the 75th percentile). The threshold multiplier a = 6.09 derives from the Rohling closed form at P_fa_ = 10^−3^. Because the 75th percentile of the exponential sits at 1.4 times the per-cell mean, OS-CFAR’s total threshold T = a·Z is approximately 10% larger than CA-CFAR’s, so the two detectors are similar in operating point, with OS-CFAR slightly being more conservative. The frame statistic is identical to CA-CFAR; reference window N_ref_ = 32, one guard frame.

### 4.5. Bounded-Memory CUSUM (Tartakovsky Variant)

The Cumulative Sum statistic [14] detects an abrupt change by accumulating per-sample log-likelihood ratios and triggering when the cumulative score exceeds a calibrated bound. The Tartakovsky variant [15,16] clips the running sum at zero from below and at K_end_ from above; both clips are practical for fixed-precision embedded implementations. The detector is fundamentally a time-domain statistic and performs no spectral decomposition. The detection threshold is calibrated from the desired per-frame false-alarm rate a_fa via h = ln(1/a_fa_); at a_fa_ = 10^−5^, h = 11.5. The per-frame increment is given in Equation (4):

Δ(m) = (X(m) − μ_0_)^2^/(2σ^2^) − (μ_1_ − μ_0_)^2^/(4σ^2^) (4)

where:

- X(m) is the broadband frame statistic of frame m;
- µ_0_ is the no-event mean of the frame statistic;
- µ_1_ is the assumed event mean;
- σ^2^ is the noise variance; all three are estimated during a 512-frame calibration window.
- h = ln(1/a_fa_) is the detection bound on the cumulative score, calibrated from the per-sample false-alarm target a_fa_ = 10^−5^, giving h = 11.5.

The increment is positive when the frame appears more consistent with the event hypothesis and negative when it appears more consistent with noise. Because CUSUM accumulates data rather than creating instant thresholds events, it is sensitive to slow systematic shifts in µ_0_: when the noise envelope drifts upward, Delta becomes positive on average and the score climbs slowly, producing a false positive. The bounded memory clipping limits, but does not eliminate, this drift sensitivity. This is a task mismatch rather than an implementation flaw: change-point detection is defined as identifying moments when the distribution of the series changes in mean or variance [15], and under the signal model of Section 3.1, the noise distribution changes continuously by design; without spectral selectivity, the detector has no basis for separating environmental change from event-driven change.

## 5. Results

This section reports the Monte Carlo campaign of Section 3.3. All values are means over five replicates per test configuration. The recommended TSNFA configuration is the hybrid variant with three-frame confirmation (Section 4.1); comparators run at their canonical published parameters (Section 2.3). The pooled TSNFA false alarm rate aggregates all frontier and scale-check test configurations, 10,800 node hours in total.

### 5.1. Headline Comparison

Table 6 summarises the headline metrics across the SNR frontier. The recommended TSNFA hybrid configuration detects 100% of events at 12 dB SNR and above, 99.5% at 5 dB, and 80.3% at 3 dB, with event precision between 99.6% and 100% in every test configuration. Eighteen false-positive clusters were observed in 10,800 node hours, a pooled rate of 0.0017 clusters per node hour (95% CI approximately 0.0010 to 0.0026). Detection latency carries the fixed 2.6 s confirmation cost stated in Section 4.1.

The comparators fall into three failure classes. CA-CFAR and OS-CFAR detect every event at every SNR but emit 42.6 and 36.8 false-positive clusters per node hour for an event precision of 2.2% and 2.6%: roughly 97 of every 100 triggers are false. The Lipski FFT detector suppresses false alarms to 1.24 clusters per node hour at 41% to 45% precision, but its fixed three-sigma threshold with the three-adjacent-bin rule detects no events below 6 dB SNR. CUSUM detects between 16% and 72% of events across the SNR range at a false alarm rate of 39 to 41 clusters per node hour. The TSNFA median variant, the per-bin rank-order formulation of the TSNFA algorithm, is reported alongside: its false alarm rate is constant at 0.005 to 0.006 clusters per node hour across the entire SNR range, but its detection rate falls to 9.6% at the bottom of the frontier.

Table 7 reports the CFAR-family configuration (10 nodes, 12 dB), which measures the tier 2 detectors of Section 2.2 under identical conditions. All four detect every event; their false alarm rates span an order of magnitude, from 5.3 clusters per node hour (TM-CFAR) to 67.6 (GO-CFAR), and precision remains at or below 15.7% for every member. The anchors within this test configuration reproduce the main campaign values (CA-CFAR 42.6, OS-CFAR 36.8, Lipski 1.24), confirming consistency with the main campaign. VI-CFAR selects its full-window CA branch on 71.4% of samples in this environment, so its operating point tracks CA-CFAR. The estimator choice therefore moves the false alarm rate but cannot rescue precision: the operating point of every family member is dominated by the shared broadband frame statistic, which is the tier design rationale of Section 2.2 and is visible in Algorithm 2, where the family members differ only in the estimator line.

### 5.2. Estimator Ablation

The variant space of Table 4 was evaluated at a matched threshold (ζ = 6.0, no confirmation frames) to isolate the effect of estimator placement. Two results are systematic. At the trigger site, the mean statistic dominates the median statistic at every SNR once thresholds are matched: the median trigger’s residual false positives arise from correlated burst pairs that survive rank-order filtering, and no threshold value separates them from low-SNR events. At the floor site, the ordering reverses: the gated median floor produces equal or fewer false positives than the EMA floor in every zero-false-positive comparison because the EMA admits slow contamination during extended disturbances that the median rejects outright. The hybrid variant is the composition of the two winners, an efficient estimator where speed of response matters and a robust estimator where contamination resistance matters. This placement is a finding of this study; the original specification [12] uses the median at both sites. Table 8 reports the ablation at the matched threshold.

Table 9 reports the threshold recalibration under the three-frame rule. 

### 5.3. Threshold Recalibration and Confirmation

Running comparators at canonical parameters (Section 2.3) raises the question whether recalibration would close the gap. Re-thresholding the recorded per-frame trigger strengths shows that it does not change the structure of the result. For the broadband detectors, detection rate and false alarm rate move together along the threshold sweep: suppressing false alarms to below 1 cluster per node hour costs essentially all detections because event energy and out-of-band interference are indistinguishable in the broadband statistic. For the Lipski detector, raising the threshold multiplier reproduces the same trade: the locked operating point (1.24 clusters per node hour, 41% to 45% precision) already sits at the favourable end of its sweep, and the price is blindness below 6 dB.

TSNFA moves differently under recalibration because confirmation decouples the per-frame threshold from the false alarm rate. Lowering ζ from 6.0 to 2.5 with M = 3 confirmation frame recovers the low-SNR detections that the unconfirmed variants miss, while the coincidence requirement keeps the confirmed false alarm rate at the pooled 0.0017 clusters per node hour of Table 6. The cost is the fixed 2.6 s confirmation latency. This is the recommended operating point.

### 5.4. Low-SNR Detection Frontier

Table 10 reports the detection-rate frontier of the recommended configuration. The transition from near zero to near one spans 1.5 to 6 dB: half of events are detected at approximately 2 dB, 80.3% at 3 dB, and the curve saturates above 6 dB. Precision remains between 99.6% and 100% along the entire frontier. This follows from the structure of the false alarm mechanism: a confirmed false positive requires noise or interference to cross the threshold on three consecutive frames, a sequence governed by the noise environment and the threshold alone, so the false alarm rate carries no dependence on event SNR. The detection rate falls with SNR, but even at the bottom of the frontier, the detector reports roughly 0.39 true events against 0.0017 false clusters per node hour, a ratio above 200:1, and precision is preserved.

**Figure 9:**
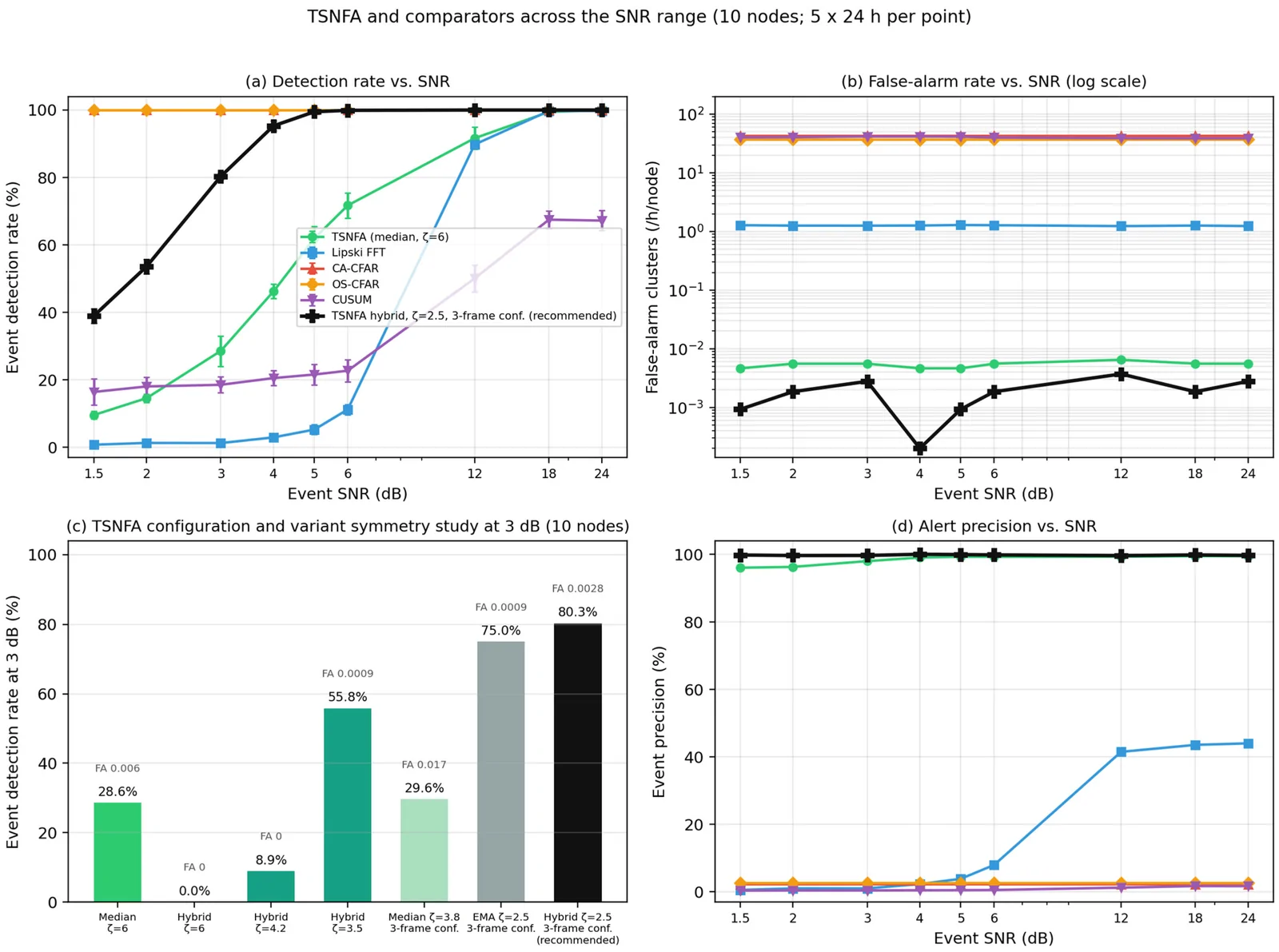
Summary of (**a**) detection rate, (**b**) precision, (**c**) false alarm rate, and (**d**) latency across the SNR frontier for all detectors.

The comparators bracket this frontier from both sides. CA-CFAR and OS-CFAR detect every event at every level, including 1.5 dB, because the broadband statistic triggers on nearly everything, which yields the false alarm rates of Table 6 as the direct consequence. The Lipski detector is the mirror image: zero detections at every level below 6 dB, then 89.8% at 6 dB rising to 99.8% at 24 dB. CUSUM rises from 16% at the bottom of the range to 72% at the top. Figure 9 summarises detection rate, precision, false alarm rate, and latency across the frontier for all detectors.

The CUSUM mechanism is SNR-dependent integrator accumulation. Under the variance-shift increment of Equation (4), these are the expected per-sample event-driven contribution scales with event power: at high SNR, the score clears the calibrated bound well within the chosen 5 s event duration (Section 3.3), while at low SNR, the score climbs more slowly, and a substantial fraction of events end before the bound is crossed. The false alarm rate is essentially SNR-invariant (39 to 41 clusters per node hour) because false alarms come from noise excursions, and the noise statistics do not change with event SNR.

### 5.5. ROC and DET Analysis

**Figure 10:**
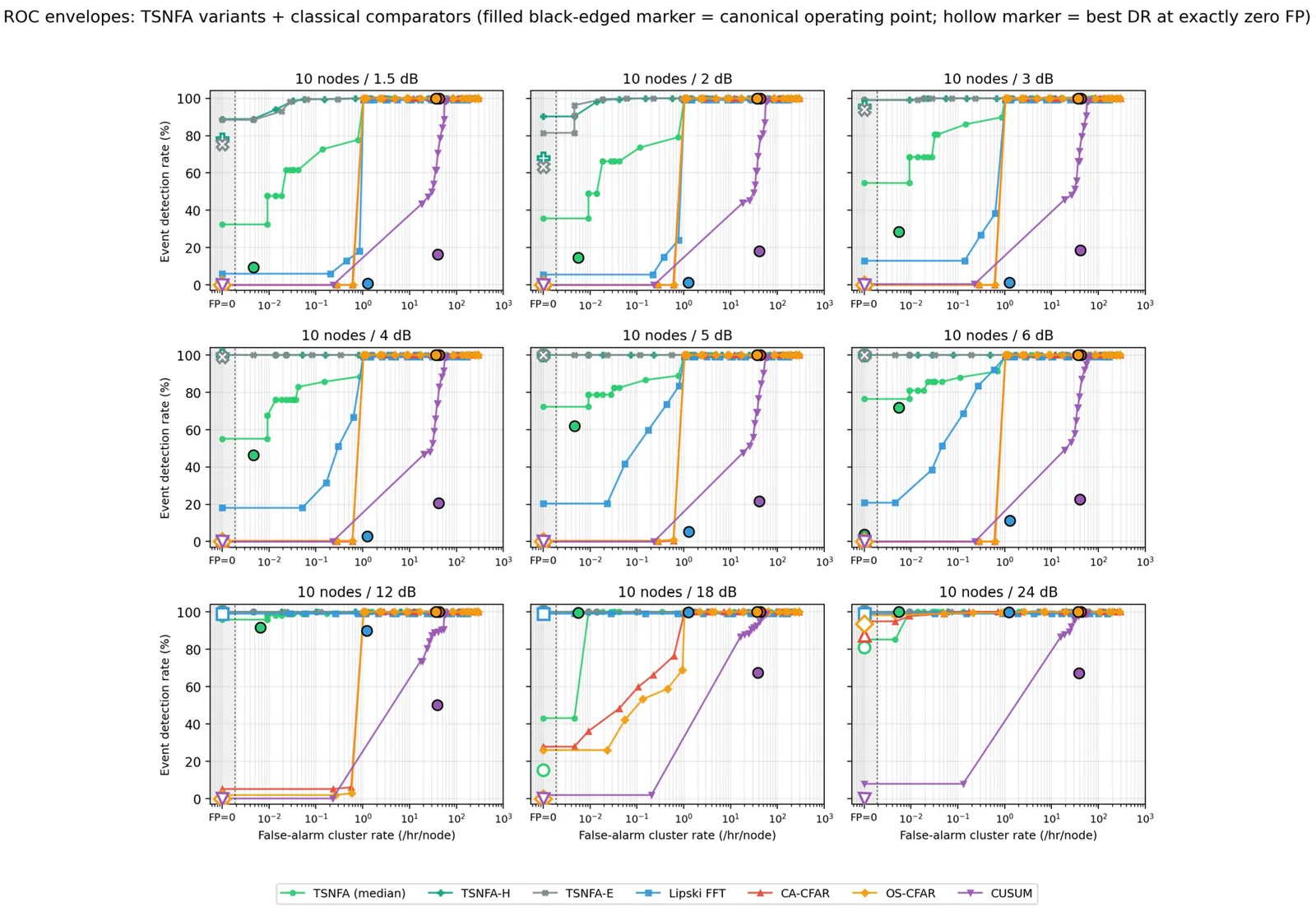
ROC envelopes per detector across the SNR frontier. Curves trace each detector’s threshold sweep; colours and marker shapes identify detectors as in the legend. The filled, black-edged marker on each curve is the detector’s operating point, the performance measured at its fixed evaluated setting under the SNR of that panel: CA-CFAR at α = 7.71, OS-CFAR at α = 6.09, the Lipski adaptation at k = 3, CUSUM at h = 11.5, and TSNFA at ζ = 2.5 with M = 3 (Section 2.3 and Section 4). The shaded column at the left of each panel holds the points with exactly zero false positives, which cannot be placed on the logarithmic axis. Hollow markers show, for orientation, the highest detection rate observed at zero false positives anywhere along the sweep; this is a selection-biased upper bound and is not used in any reported result.

**Figure 11:**
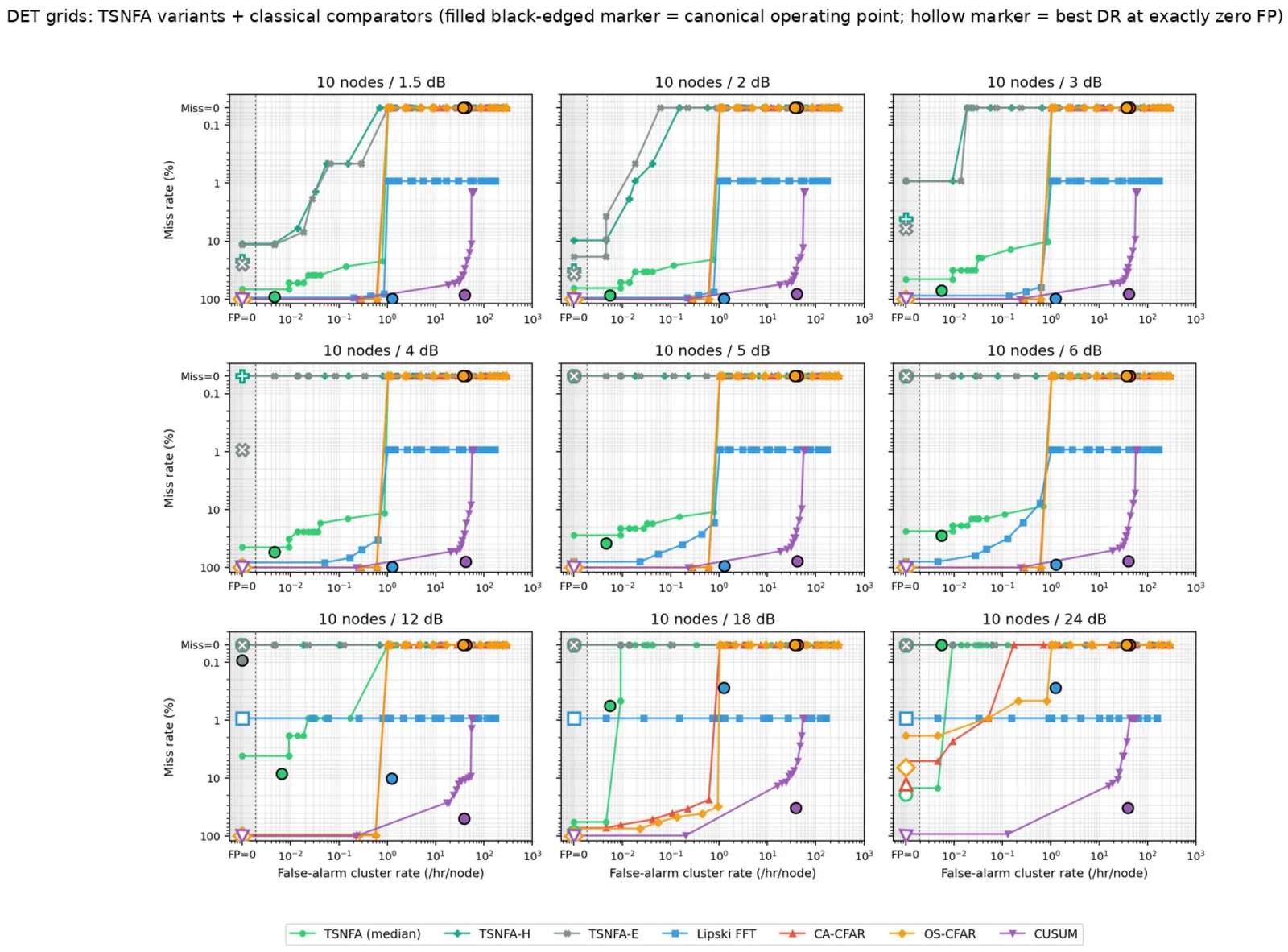
DET curves [34] for the frontier test configurations at 10 nodes. Each detector’s operating point is marked, showing the performance measured at its fixed evaluated setting under the SNR of that panel: CA-CFAR at α = 7.71, OS-CFAR at α = 6.09, the Lipski adaptation at k = 3, CUSUM at h = 11.5, and TSNFA at ζ = 2.5 with M = 3. Axes are scaled to resolve the low-false alarm region where the ROC of Figure 10 saturates. Hollow markers show, for orientation, the highest detection rate observed at zero false positives anywhere along the threshold sweep; this is a selection-biased upper bound and is not used in any reported result.

Receiver operating characteristic (ROC) and detection error trade-off (DET) [34] analyses are read per threshold and per replicate from the recorded trigger strengths. The ROC envelopes separate the detectors into the same three classes as Table 6, and the DET view resolves the low-false alarm corner where the ROC saturates (Figure 10 and Figure 11).

### 5.6. Bandwidth and Scaling

Per-node report traffic is proportional to the sum of true and false event clusters. At one scheduled event per node hour, the recommended configuration transmits approximately one cluster report per node hour (1.0 true + 0.0017 false); CA-CFAR and OS-CFAR transmit 43.6 and 37.8 (a factor of roughly 40 more, of which more than 97% is false); the Lipski detector transmits 2.2; and CUSUM transmits 40 to 42, dominated by false clusters. The false-positive report traffic from CA-CFAR and OS-CFAR therefore exceeds that of the recommended TSNFA configuration by more than four orders of magnitude, and this ratio is independent of the byte-level encoding of reports. Per-node report rates are given in Table 11.

The 50-node scale checks confirm that per-node detection statistics survive the transition from the sparse to the dense regime (Section 3.3): detection rate at 3 dB moves from 80.3% to 79.5%, and the false alarm rate at 50 nodes is 0.0044 clusters per node hour against the pooled 0.0017. Both remain three or more orders of magnitude below the broadband comparators. Network detection latency grows with mean hop count as the mesh scales; the fixed 2.6 s confirmation latency of the recommended configuration adds to the network latency and is reported separately. The scale checks are summarised in Table 12.

### 5.7. Cortex-M0+ Resource Impact at Network Level

Section 3.5 established that the detector compute is not the binding resource: the most expensive variant costs 272 mJ/h of MCU energy, while a single LoRa report (SF9, 20 B payload, 185.3 ms time-on-air) costs 27.5 mJ. Implementation cost of CFAR detectors has previously been characterised by FPGA, CPU, and GPU class platforms [35]; MCU-class deployments have not been studied, which motivated the measurements of Section 3.5. The binding resource is the radio link. Under the 1% duty-cycle limit of the EU868 band, the airtime budget is 36 s per node hour; at 42.6 false clusters per node hour, CA-CFAR consumes at least 7.9 s of that budget on false reports before any relay multiplication, and multi-hop forwarding in the dense regime multiplies this load across the mesh. False-positive suppression, not code optimisation, therefore determines both the schedulability of the radio and node battery life. Single-hop radio-cost bounds are given in Table 13.

## 6. Discussion

### 6.1. What the Simulation Does and Does Not Establish

A Monte Carlo simulation spanning nine SNR levels, two network sizes, and a supplementary comparison of four further CFAR detectors is evidence, not proof. Under the signal model of Section 3 and the evaluated detector settings, the simulation shows that TSNFA sustains high detection, high precision, and low false alarm load simultaneously and that no classical comparator does. The signal model is realistic in that it incorporates time-varying noise power, mains harmonics, and digital switching bursts at amplitudes consistent with deployed hardware measurements, but it remains a model. The simulation is therefore most useful for a comparative claim and least useful for an absolute claim; deployed hardware results in [12] support the absolute claim independently.

### 6.2. The CFAR-Family Interpretation

Section 2 framed TSNFA as a CFAR variant adapted to a 1D temporal stream rather than a 2D range–Doppler matrix, and Section 5 found that both CA-CFAR and OS-CFAR settle into essentially the same operating point, dominated not by the difference between mean and rank statistics but by their shared use of the broadband-energy frame statistic. Both detectors were designed assuming an exponentially distributed cell statistic. Our hardware-constrained adaptation replaces that per-cell quantity with a sum of over 128 squared samples; by the central limit theorem, the cell statistic is no longer exponential but approximately Gaussian. The Rohling and Finn-Johnson threshold formulas are calibrated for the exponential cell distribution; applied to the Gaussian sum, they overshoot the bulk by = 54 standard deviations, and the empirical false alarm rate is determined by non-Gaussian heavy-tail interference for which neither detector provides spectral rejection. TSNFA addresses the same hardware constraint at the FFT step rather than at the threshold: by computing per-bin |X_k_|^2^, it recovers a chi-squared-with-two-degrees-of-freedom (exponential) cell statistic per bin, on which a multiplicative threshold operates correctly.

### 6.3. The Bandwidth Dimension

Classical CFAR papers do not report communication cost, because in radar, the detector output never crosses a constrained link: detection and processing sit in the same receiver. In an IoT mesh, every detection decision must be transmitted so the report traffic a detector generates is as much a performance metric as its detection rate. The decision-relevant quantity is the false-positive report rate. At one scheduled event per node hour, CA-CFAR and OS-CFAR transmit roughly 40 times the cluster reports of the recommended TSNFA configuration (43.6 and 37.8 against 1.0 per node hour, Table 11), with more than 97% of them being false, and their false-positive report traffic alone exceeds TSNFA’s by more than four orders of magnitude (Section 5.6). The Lipski adaptation is bandwidth-comparable to TSNFA (2.2 against 1.0 cluster reports per node hour, Table 11), so its deployment cost appears elsewhere: it detects nothing below 6 dB SNR. The two detectors load the radio similarly; they differ in which events they detect. On the resource side, Section 5.7 shows that the false-positive load, not detector compute, is what binds the LoRa duty-cycle budget and node battery life. The textbook CFAR figure of merit (probability of detection at a calibrated probability of false alarm) is therefore necessary but not sufficient for IoT applications: the report traffic that a detector’s false alarms impose on the mesh is a deployment criterion of equal weight.

### 6.4. Threats to Validity

Five threats to validity deserve acknowledgement. The noise model is synthetic; real environments produce correlated, non-stationary, and event-like noise that the generator does not capture. The drift cycle is fixed at 1 hour with ±6 dB excursion; slower drift would advantage detectors with longer adaptation time constants. The event waveform is a single fixed shape and duration (damped sinusoid in 1–5 Hz; the chosen 5 s event duration of Section 3.3); real perimeter-security events span a wider range, and a detector tuned to one waveform may not generalise to others. A fourth threat: all five detectors run on the same simulator against the same input realisation at each Monte Carlo seed, which eliminates inter-detector variance but means a simulator peculiarity would influence all detectors in correlated ways. A fifth threat is internal: the Lipski adaptation applies a Hann window before the FFT, while TSNFA does not. The window is an implementation setting of the adaptation, fixed in the deployed system alongside k = 3 (Section 4.2), not a published Lipski parameter: the published detector operates in the time domain and applies no frame window. The asymmetry is small: a processing loss of approximately 1.76 dB, equivalently a 50% increase in noise bandwidth per bin, but it is a real difference. The two FFT-based detectors do not use the same frame window: the Lipski adaptation tapers each 128-sample frame with a Hann window before the FFT, while TSNFA transforms the raw frame. Equalising this, running both detectors with the same window, either Hann for both or none for both, would remove the asymmetry, and we chose not to, for two reasons. First, both implementations were locked and verified before the comparison, and modifying either afterwards would reopen that verification. Second, the difference has no clear winner: the Hann window helps the Lipski adaptation by suppressing spectral leakage, so less out-of-band interference smears into the event band, but it also costs approximately 1.76 dB of per-bin signal margin, which hurts exactly where that detector is weakest, near its 6 dB sensitivity limit. An asymmetry bounded near 1.76 dB, with an ambiguous net sign, cannot account for performance differences measured in orders of magnitude.

## 7. Conclusions

We presented a Monte Carlo comparison of TSNFA against canonical detectors drawn from the radar CFAR family and from sequential change detection, evaluated under hardware constraints representative of Cortex-M0+ class IoT sensor nodes, across a nine-level SNR frontier with scale checks at 50 nodes and a supplementary configuration covering four further CFAR-family detectors. The recommended TSNFA configuration, a mean-trigger, gated-median-floor hybrid with three-frame confirmation selected by estimator ablation, detects 100% of events at 12 dB SNR and above, 99.5% at 5 dB, and 80.3% at 3 dB, with 99.6% to 100% event precision and a pooled false-alarm rate of 0.0017 clusters per node hour at a fixed confirmation cost of 2.6 s of detection latency. No comparator matches this combination: CA-CFAR and OS-CFAR detect every event but at 42.6 and 36.8 false clusters per node hour (precision below 3%), the Lipski FFT detector reaches 1.24 clusters per node hour but detects nothing below 6 dB, and CUSUM detects 16% to 72% of events across the range. In report traffic, the false-positive load of CA-CFAR and OS-CFAR exceeds that of the recommended TSNFA configuration by more than four orders of magnitude, and the resource measurements of Section 3.5 show that this false-positive load, not detector compute, is what binds the radio duty cycle and battery life of a LoRa-class node. The contribution is best read as a development within the CFAR family rather than a departure from it. CA-CFAR and OS-CFAR were formulated for 2D range–Doppler matrices in which each cell is the squared magnitude of a single complex Fourier sample and is therefore exponentially distributed; transposed to a broadband single-node frame statistic, that cell distribution assumption fails, and the calibrated threshold multipliers lose their meaning. TSNFA restores the assumption by thresholding per-bin spectral magnitudes, to which the textbook exponential-tail calibration applies and adds the band-selection, persistence, floor-gating, and confirmation stages that the single-channel temporal setting requires. The estimator ablation also yields a simple design rule: use a fast estimator where the detector must respond quickly, and a robust one where it must resist contamination. In TSNFA, this means the mean at the trigger so a genuine event registers within the three-frame confirmation span and the gated median registers for the noise floor, so interference cannot raise the threshold. Two practices from this study carry over to comparative detector work in general. First, fix and verify every comparator implementation against its published results before running the comparison. Second, compute ROC and DET curves within each replicate before combining them; picking the best value across pooled replicates makes every detector look better than it is.

## Figures and Tables

**Table 1:** Configuration matrix.

Test Configuration	Nodes	SNR (dB)	Duration	MC Runs	Events (Mean)
SNR frontier	10	1.5, 2, 3, 4, 5, 6, 12, 18, 24	24 h	5 per level	≈240/run
Scale checks	50	3, 12, 18	24 h	5 per level	≈1180/run
CFAR-family configuration (GO, SO, TM, VI)	10	12	24 h	5	≈240/run

**Table 2:** STM32G030F6 resource usage per frame (128 samples, Tb = 1.28 s, fCPU = 8 MHz, HSI). Radio airtime and transmit energy per detector are reported in the Results, computed from the network loads of Section 5.6.

Algorithm	ΔFlash, kB	ΔSRAM, B	t, ms	CPU, %	E(MCU), mJ/h
TSNFA, 128-pt FFT Q15 (as published)	≈1.7	2380	10.09	0.79	272
TSNFA, Goertzel bank	≈0.8	1868	8.33	0.65	225
TSNFA, Goertzel + incremental median	≈0.9	3160	1.71	0.13	46
Lipski FFT (k = 3)	≈1.8	≈1100	2.90	0.23	78
CA-CFAR	≈0.30	160	0.12	0.010	3.2
OS-CFAR	≈0.45	288	0.33	0.025	8.9
CUSUM	≈0.35	48	0.15	0.012	4.1

**Table 3:** TSNFA parameters.

Parameter	Symbol	Value	Reasoning
FFT size	L	128	Delta f = 0.78 Hz at f_s_ = 100 Hz
Event band	[f_low, f_high]	[1,5] Hz	Bins k in {1, …, 6}
Trigger depth	γ_d_	3	Tolerates 1 outlier in 3
Floor depth	γ_a_	64	Tolerates 31 outliers in 64; tau = 82 s
Threshold multiplier	ζ	2.5 (hybrid); 6.0	Calibrated per Section 4.1
Confirmation frames	M	3	Binary integration [13]; +2.6 s latency
Floor-update hysteresis	rho	0.8	Floor updates only when ratio < rho
Trigger logic	-	OR across bins [M]; max-bin scalar path [E,H]	Equivalent to OR across bins

**Table 4:** TSNFA variant definitions. Variants differ at the two estimator sites, the band topology (per bin against max-across-bins), the threshold multiplier, and the confirmation rule. All floor updates are gated (Section 4.1). The “*median variant*” uses *S_k_* and *N_k_*, the “*mean variant*” *S* and *N*, and the “*hybrid variant*” uses *S_k_* and *N*.

Variant	Trigger Statistic (*S_k_* or *S*)	Noise Floor (*N_k_* or *N*)	ζ	Confirmation
Median (as specified [12])	per-bin median over 3 frames	per-bin gated median(64)	6.0	none
Mean (EMA)	mean over 3 frames of max-across-bins	gated EMA (alpha = 1 − 1/64)	6.0	none
Hybrid (recommended)	mean over 3 frames of max-across-bins	gated median(64), single floor	2.5	3 consecutive frames

**Table 5:** Lipski FFT parameters.

Reasoning	Value	Symbol	Parameter
Three-sigma default (adaptation)	3	k	Threshold multiplier
Spectral persistence	3	N_bins_min_	Min adjacent bins above
≈128 s of noise-only	100 frames	M_cal_	Calibration window
EMA on no-detection frames	0.01	α	Slow-update coefficient
Reduces spectral leakage	Hann	—	Window function

**Table 6:** Headline metrics across the SNR frontier (10 nodes; Section 3.3). TSNFA false alarm rate is pooled over all test configurations (10,800 node hours).

Detector	DR (%), 1.5–24 dB	Precision (%)	FA (cl/h/Node)	Note
TSNFA hybrid (recommended)	39.0–100	99.6–100	0.0017 (pooled)	100% at ≥12 dB; 80.3% at 3 dB
TSNFA median (as specified)	9.6–100	96.0–99.5	0.005–0.006	FA rate constant across SNR
Lipski FFT (k = 3)	0–99.8	41–45	1.24	no detections below 6 dB
CA-CFAR	100 at all SNRs	2.2	42.6	broadband statistic
OS-CFAR	100 at all SNRs	2.6	36.8	broadband statistic
CUSUM	16–72	<2	39–41	SNR-dependent accumulation

**Table 7:** The CFAR family configuration at 10 nodes, 12 dB SNR (tier 2 of Section 2.2).

Detector	DR (%)	Precision (%)	FA (cl/h/Node)	Note
GO-CFAR [6]	100	1.5	67.6	greatest-of selection
SO-CFAR [5]	100	3.4	28.6	smallest-of selection
TM-CFAR [7]	100	15.7	5.3	trimming saturates in this regime
VI-CFAR [10]	100	1.5	66.6	CA branch on 71.4% of samples

**Table 8:** Estimator ablation at the matched threshold (10 nodes, 12 dB, ζ = 6.0, no confirmation frames). The hybrid composition of the two winning estimators is evaluated at its recommended operating point in Table 6 and Table 9.

Variant (ζ = 6.0, M = 1)	DR at 12 dB (%)	FA (cl/h/Node)	Dominant Residual Error
Median trigger, median floor	91.3	0.005–0.006	correlated burst pairs, threshold-inseparable from low-SNR events
Mean trigger, EMA floor (deployed v1.1)	100.0	0 observed in 1200 node-h	floor contamination under extended disturbances (loses every zero-FP floor comparison to the median)

**Table 9:** Threshold recalibration with confirmation: lowering ζ from 6.0 to 2.5 under the three-frame rule raises the low-SNR frontier while reducing the false-alarm rate.

Operating Point	ζ	M	DR (%), 1.5–24 dB	DR at 12 dB (%)	FA (cl/h/Node)	Precision (%)	Added Latency
median variant, no confirmation frames	6.0	1	9.6–100	91.3	0.005–0.006	96.0–99.5	none
Hybrid, 3 confirmation frames (recommended)	2.5	3	39.0–100	100	0.0017 (pooled)	99.6–100	2.6 s

**Table 10:** Low-SNR detection frontier of the recommended configuration (10 nodes, five replicates per level).

SNR (dB)	1.5	2	3	4	5	6	12	18	24
TSNFA hybrid DR (%)	39.0	53.6	80.3	95.3	99.5	99.9	100	100	100

**Table 11:** Per-node cluster report rates at one scheduled event per node hour, computed from the measured detection and false alarm rates of Table 6. Byte-level network load scales linearly with the report encoding and is independent of the ratios shown.

Detector	True Reports (/h/Node)	False Reports (/h/Node)	Total (/h/Node)	False Share (%)
TSNFA hybrid (recommended)	1.00	0.0017	1.00	0.17
Lipski FFT (≥6 dB)	0.90–1.00	1.24	2.2	55–58
CA-CFAR	1.00	42.6	43.6	97.7
OS-CFAR	1.00	36.8	37.8	97.4
CUSUM	0.16–0.72	39–41	~40	>98

**Table 12:** Scale checks: recommended configuration at 10 versus 50 nodes.

Metric	10 Nodes	50 Nodes
Detection rate at 3 dB (%)	80.3	79.5
False-alarm rate (cl/h/node)	0.0017 (pooled)	0.0044

**Table 13:** Single-hop lower bounds on radio cost per node, from the report rates of Table 11 at 185.3 ms and 27.5 mJ per LoRa report (SF9, 20 B payload) against the 36 s per node-hour EU868 duty-cycle budget. Multi-hop relay traffic in the dense regime multiplies these values across the mesh; per-detector MCU energy (3.2 to 272 mJ/h, Table 2) is small against the comparator radio cost.

Detector	Reports (/h/Node)	Airtime (s/h)	Duty-Cycle Share (%)	E(Radio) (J/h)
TSNFA hybrid (recommended)	1.00	0.19	0.5	0.028
Lipski FFT	2.2	0.42	1.2	0.062
CA-CFAR	43.6	8.1	22	1.20
OS-CFAR	37.8	7.0	19	1.04
CUSUM	~40	7.4	21	1.10

## Data Availability

The data presented in this study, including the simulator source code, configuration files, and per-frame trigger-strength traces, are openly available at Zenodo at 10.5281/zenodo.22003555 (reference [32]) (accessed on 28 August 2026). An interactive data page is available at https://github.com/Gnacode/MDPI-MONTECARLO (accessed on 28 August 2026).

## Supplementary Materials

The complete simulator source code, configuration files, and per-frame strength traces from which the ROC and DET curves of Figure 10 and Figure 11 are derived are permanently archived at Zenodo [32]. An interactive version of the data page, with hover-resolved exact values and per-replicate distributions for all figures, is hosted at https://github.com/Gnacode/MDPI-MONTECARLO, (accessed on 28 August 2026).

## Abbreviations

The following abbreviations are used in this manuscript:

ADC	Analog-to-Digital Converter
CA-CFAR	Cell-Averaging Constant False Alarm Rate
CFAR	Constant False Alarm Rate
CSMA/CA	Carrier-Sense Multiple Access with Collision Avoidance
CUSUM	Cumulative Sum
DR	Detection Rate
EMA	Exponential Moving Average
EMI	Electromagnetic Interference
FAR	False Alarm Rate
FFT	Fast Fourier Transform
FP	False Positive
IoT	Internet of Things
LoRa	Long Range (radio)
MC	Monte Carlo
OS-CFAR	Ordered-Statistic Constant False Alarm Rate
ROC	Receiver Operating Characteristic
SNR	Signal-to-Noise Ratio
TP	True Positive
TSNFA	Temporal Spectral Noise-Floor Adaptation

## References

[1] Pioli L., Dorneles C.F., de Macedo D.D.J., Dantas M.A.R. (2022). An overview of data reduction solutions at the edge of IoT systems: A systematic mapping of the literature. Computing.

[2] Pioli L., de Macedo D.D.J., Costa D.G., Dantas M.A.R. (2024). Intelligent Edge-powered Data Reduction: A Systematic Literature Review. ACM Comput. Surv..

[3] Finn H.M., Johnson R.S. (1968). Adaptive detection mode with threshold control as a function of spatially sampled clutter level estimates. RCA Rev..

[4] Rohling H. (1983). Radar CFAR thresholding in clutter and multiple target situations. IEEE Trans. Aerosp. Electron. Syst..

[5] Trunk G.V. (1978). Range resolution of targets using automatic detectors. IEEE Trans. Aerosp. Electron. Syst..

[6] Hansen V.G., Sawyers J.H. (1980). Detectability Loss Due to “Greatest Of” Selection in a Cell-Averaging CFAR. IEEE Trans. Aerosp. Electron. Syst..

[7] Gandhi P.P., Kassam S.A. (1988). Analysis of CFAR processors in nonhomogeneous background. IEEE Trans. Aerosp. Electron. Syst..

[8] Rickard J.T., Dillard G.M. (1977). Adaptive Detection Algorithms for Multiple-Target Situations. IEEE Trans. Aerosp. Electron. Syst..

[9] Himonas S.D., Barkat M. (1992). Automatic censored CFAR detection for nonhomogeneous environments. IEEE Trans. Aerosp. Electron. Syst..

[10] Smith M.E., Varshney P.K. (2000). Intelligent CFAR processor based on data variability. IEEE Trans. Aerosp. Electron. Syst..

[11] Subramanyan N.R., Kalpathi Rajendran R., Vengadarajan A. (2019). Robust variability index CFAR for non-homogeneous background. IET Radar Sonar Navig..

[12] Makovetskyi S., Thomsen L. (2026). Temporal Spectral Noise-Floor Adaptation for Error-Intolerant Trigger Integrity in IoT Mesh Sensor Networks. IEEE Internet Things J..

[13] Schwartz M. (1956). A coincidence procedure for signal detection. IRE Trans. Inf. Theory.

[14] Page E.S. (1954). Continuous inspection schemes. Biometrika.

[15] Torre A., Taylor A., Poullin D., Chonavel T. Parameters Extraction of Unknown Radar Signals Using Change Point Detection. Proceedings of the 2023 IEEE International Radar Conference (RADAR 2023).

[16] Tartakovsky A.G., Polunchenko A.S., Sokolov G. (2013). Efficient computer network anomaly detection by changepoint detection methods. IEEE J. Sel. Top. Signal Process..

[17] Trilles S., Hammad S.S., Iskandaryan D. (2024). Anomaly detection based on Artificial Intelligence of Things: A Systematic Literature Mapping. Internet Things.

[18] Abadade Y., Temouden A., Bamoumen H., Benamar N., Chtouki Y., Hafid A.S. (2023). A Comprehensive Survey on TinyML. IEEE Access.

[19] Tsoukas V., Gkogkidis A., Boumpa E., Kakarountas A. (2024). A Review on the emerging technology of TinyML. ACM Comput. Surv..

[20] Capogrosso L., Cunico F., Cheng D.S., Fummi F., Cristani M. (2024). A Machine Learning-Oriented Survey on Tiny Machine Learning. IEEE Access.

[21] Mahmoud Q.H. (2025). Tiny Machine Learning and On-Device Inference: A Survey of Applications, Challenges, and Future Directions. Sensors.

[22] Hammad S.S., Iskandaryan D., Trilles S. (2023). An unsupervised TinyML approach applied to the detection of urban noise anomalies. Internet Things.

[23] Antonini M., Pincheira M., Vecchio M., Antonelli F. (2023). An Adaptable and Unsupervised TinyML Anomaly Detection System for Extreme Industrial Environments. Sensors.

[24] Arciniegas S., Rivero D., Pinan J., Diaz E., Rivas F. (2025). IoT device for detecting abnormal vibrations in motors using TinyML. Discov. Internet Things.

[25] Liu K., Li Y., Wang P. (2023). A CFAR Detection Algorithm Based on Clutter Knowledge for Cognitive Radar. IEICE Trans. Fundam..

[26] Shen L., Su H., Mao Z. (2023). Signal Property Information-Based Target Detection with Dual-Output Neural Network in Complex Environments. Sensors.

[27] Kang S., Jang M., Lee S. (2022). Autoencoder-Based Target Detection in Automotive MIMO FMCW Radar System. Sensors.

[28] Akhtar J. (2021). A neural network framework for binary classification of radar detections. EURASIP J. Adv. Signal Process..

[29] Lipski M.V., Kompella S., Narayanan R.M. (2021). Practical implementation of adaptive threshold energy detection using software defined radio. IEEE Trans. Aerosp. Electron. Syst..

[30] Kalyan B., Balasuriya A. Sonar based automatic target detection scheme for underwater environments using CFAR techniques: A comparative study. Proceedings of the 2004 International Symposium on Underwater Technology.

[31] Yang B., Zhang H. (2022). A CFAR Algorithm Based on Monte Carlo Method for Millimeter-Wave Radar Road Traffic Target Detection. Remote Sens..

[32] Thomsen L. (2026). TSNFA Monte Carlo Simulation: Code and Figures–Gnacode/MDPI-MONTECARLO: Revised.

[33] Cooley J.W., Tukey J.W. (1965). An algorithm for the machine calculation of complex Fourier series. Math. Comput..

[34] Martin A., Doddington G., Kamm T., Ordowski M., Przybocki M. The DET curve in assessment of detection task performance. Proceedings of the Eurospeech 1997.

[35] Bales M.R., Benson T., Dickerson R., Campbell D., Hersey R., Culpepper E. Real-time implementations of ordered-statistic CFAR. Proceedings of the 2012 IEEE Radar Conference.

